# Essential Role of Somatic Kv2 Channels in High-Frequency Firing in Cartwheel Cells of the Dorsal Cochlear Nucleus

**DOI:** 10.1523/ENEURO.0515-20.2021

**Published:** 2021-05-04

**Authors:** Tomohiko Irie

**Affiliations:** Division of Pharmacology, National Institute of Health Sciences, Kawasaki City, Kanagawa 210-9501, Japan

**Keywords:** cartwheel cells, dorsal cochlear nucleus, guangxitoxin-1E, Kv2 channels, sustained firing

## Abstract

Among all voltage-gated potassium (Kv) channels, Kv2 channels are the most widely expressed in the mammalian brain. However, studying Kv2 in neurons has been challenging because of a lack of high-selective blockers. Recently, a peptide toxin, guangxitoxin-1E (GxTX), has been identified as a specific inhibitor of Kv2, thus facilitating the study of Kv2 in neurons. The mammalian dorsal cochlear nucleus (DCN) integrates auditory and somatosensory information. In the DCN, cartwheel inhibitory interneurons receive excitatory synaptic inputs from parallel fibers conveying somatosensory information. The activation of parallel fibers drives action potentials in the cartwheel cells up to 130 Hz *in vivo*, and the excitation of cartwheel cells leads to the strong inhibition of principal cells. Therefore, cartwheel cells play crucial roles in monaural sound localization and cancelling detection of self-generated sounds. However, how Kv2 controls the high-frequency firing in cartwheel cells is unknown. In this study, we performed immunofluorescence labeling with anti-Kv2.1 and anti-Kv2.2 antibodies using fixed mouse brainstem slice preparations. The results revealed that Kv2.1 and Kv2.2 were largely present on the cartwheel cell body membrane but not on the axon initial segment (AIS) nor the proximal dendrite. Whole-cell patch-clamp recordings using mouse brainstem slice preparation and GxTX demonstrated that blockade of Kv2 induced failure of parallel fiber-induced action potentials when parallel fibers were stimulated at high frequencies (30–100 Hz). Thus, somatic Kv2 in cartwheel cells regulates the action potentials in a frequency-dependent manner and may play important roles in the DCN function.

## Significance Statement

The mammalian dorsal cochlear nucleus (DCN) plays a role in the monaural sound localization and cancelling detection of self-generated sounds. In the DCN, cartwheel cells receive excitatory synaptic inputs from parallel fibers. Parallel fiber activation can drive action potentials in cartwheel cells at high frequency, but the ionic mechanism of such firing remains unknown. In this study, we found that voltage-gated potassium (Kv)2.1 and Kv2.2 ion channels were present on the cell bodies of cartwheel cells. Application of a specific blocker of Kv2 induced failure of parallel fiber-induced action potentials only when presynaptic parallel fibers were stimulated at high frequencies. Thus, somatic Kv2 in cartwheel cells regulates action potentials in a frequency-dependent manner and may play important roles in sound processing.

## Introduction

Neurons express a wide variety of voltage-gated potassium (Kv) channels that feature different voltage dependence and kinetics, thereby endowing neurons with a wide range of firing patterns (Hille, 2001; Vacher et al., 2008; Johnston et al., 2010). The Kv channel family can be divided into several subfamilies based on nucleotide sequence similarity and function. In the mammalian brain, messenger RNA expression studies and antibody-based protein detection indicate that Kv2 channels are the most widely expressed voltage-gated K channels in terms of tissue distribution (Trimmer, 1991; Hwang et al., 1993; Mandikian et al., 2014). Kv2 channels consist of Kv2.1 and Kv2.2, and are present not only in cortical, hippocampal, and α-motoneurons but also in retinal bipolar cells, cardiac myocytes, vascular and gastrointestinal smooth muscle, and pancreatic β cells (Johnson et al., 2019). Physiologically, Kv2 channels produce delayed-rectifier currents, and Kv2.1 channels have a high activation threshold of –15 mV (Johnston et al., 2010; Johnson et al., 2019). Indeed, delayed-rectifier currents in various neurons are generally because of Kv2 channels (Murakoshi and Trimmer, 1999; Malin and Nerbonne, 2002; Guan et al., 2007; Johnston et al., 2008; Tong et al., 2013). However, the study of Kv2-mediated current in neurons has been challenging because of the limited availability of high-selective blockers. In previous studies, Kv2 channels were blocked by an anti-Kv2 antibody, gene expression of dominant-negative Kv2 channels, or non-specific Kv2 channel blockers, or were eliminated by the knock-out mice. In 2006, a peptide toxin, guangxitoxin-1E (GxTX), extracted from the venom of the Chinese tarantula *Plesiophrictus guangxiensis*, has been identified as a potent and specific inhibitor of Kv2.1 and Kv2.2 channels, with a half-blocking concentration of 2–5 nm (Herrington et al., 2006; Herrington, 2007). Using this toxin, the physiological roles of Kv2 channels have been explored in the superior cervical ganglion neurons, CA1 pyramidal neurons, and the entorhinal cortex layer II stellate cells (Liu and Bean, 2014; Kimm et al., 2015; Hönigsperger et al., 2017). Interestingly, Kv2.1 expression in some types of cerebral neurons coincides with the presence of clustered ryanodine receptors in the cell body, suggesting that Kv2.1 expression can be observed in neurons expressing clustered ryanodine receptors in other brain regions such as the brainstem (Mandikian et al., 2014).

The mammalian brainstem contains several auditory nuclei, and among them, the dorsal cochlear nucleus (DCN) integrates auditory inputs and multimodal ones, including somatosensory, vestibular, and higher-level auditory information (Oertel and Young, 2004). Auditory inputs are conveyed by auditory nerve fibers and multimodal inputs by parallel fibers, which are axons of excitatory granule cells in the cochlear nuclei. The DCN plays several crucial roles in the monaural sound localization and cancelling detection of self-generated sounds (e.g., sound induced by licking behavior; May, 2000; Singla et al., 2017). Singla et al. (2017) reported that during licking behavior in mice, complex-spiking single units (i.e., firing brief high-frequency bursts of spikes) were activated in the DCN; these complex-spiking neurons likely correspond to cartwheel inhibitory interneurons (Manis et al., 1994; Davis and Young, 1997; Tzounopoulos et al., 2004; Roberts et al., 2008; Ma and Brenowitz, 2012). Cartwheel cells are medium-sized GABAergic/glycinergic neurons which fire mixtures of simple and complex action potentials (Manis et al., 1994; Golding and Oertel, 1996; Kim and Trussell, 2007; Roberts et al., 2008; Roberts and Trussell, 2010). Moreover, cartwheel cells have profuse spiny dendrites that receive excitatory synaptic inputs from parallel fibers (Wouterlood and Mugnaini, 1984). The activation of parallel fibers drives action potentials in cartwheel cells up to 130 Hz *in vivo*, and the excitation of cartwheel cells leads to the strong inhibition of principal cells (Davis and Young, 1997; Roberts and Trussell, 2010). Recently, it has been shown that cartwheel cells express clustered ryanodine receptors abundantly at putative subsurface cisterns immediately beneath the somatic membrane (Yang et al., 2016; Irie and Trussell, 2017). Considering that clustered ryanodine receptor-expressing neurons have Kv2.1 channels (Mandikian et al., 2014), it is possible that cartwheel cells also express Kv2.1 channels. However, where Kv2 channels are expressed in cartwheel cells in terms of subcellular localization and how Kv2 channels control high-frequency firing are unknown.

In this study, we performed immunofluorescence labeling using anti-Kv2.1, anti-Kv2.2 channel, and other antibodies in fixed mouse brainstem slice preparations. We also performed electrophysiological recordings from cartwheel cells in acute brainstem slice preparations using the whole-cell patch-clamp method and GxTX, a specific Kv2 channel blocker. Immunofluorescence labeling revealed that Kv2.1 and Kv2.2 channels largely localized on the membrane of the cell body, not at the axon initial segment (AIS) nor the dendrite. Furthermore, blocking Kv2 channels by GxTX induced failures of parallel fiber-induced action potential generations only when parallel fibers were stimulated at high frequency (30–100 Hz). Thus, somatic Kv2 channels regulate action potentials in a frequency-dependent manner in cartwheel cells.

## Materials and Methods

### Animals

All animal care and handling procedures used in this study were approved by the Institutional Animal Care and Use Committee. Two male (age: 27 and 30 d) and one female (age: 34 d) ICR mice were used for fluorescence immunohistochemistry. ICR mice of both sexes (81 animals; age: 21–34 d) were used for electrophysiological recordings.

### Fluorescence immunohistochemistry and confocal imaging

Mice were deeply anesthetized with isoflurane and fixed by transcardial perfusion of PBS, followed by 4% (wt/vol) ice-cold formaldehyde in PBS. The brains were removed from skulls, postfixed in formaldehyde at room temperature for 30 min, and cryoprotected in 30% (w/w) sucrose-containing PBS overnight at 4°C. Using a cryostat (CM3050S, Leica Microsystems), the cerebral hemispheres and brainstems were sliced into 30-μm-thick coronal sections. The sections were washed with PBS containing 0.3% (vol/vol) Triton X-100 (PBS-X), and then incubated overnight at 4°C in incubation buffer [1% (vol/vol) goat and donkey serum, 0.25% (wt/vol) λ-carrageenan, and 0.02% (wt/vol) sodium azide in PBS-X] containing the following four kinds of primary antibodies: mouse IgG2a monoclonal anti-ankyrin-G antibody (1 μg/ml, clone N160/36, UC Davis/NIH NeuroMab Facility; King et al., 2014; Yang et al., 2016), mouse IgG3 monoclonal anti-Kv2.1 antibody (1 μg/ml, clone L80/21, UC Davis/NIH NeuroMab Facility; Bishop et al., 2015), mouse IgG2b monoclonal anti-Kv2.2 antibody (1 μg/ml, clone N372B/60, UC Davis/NIH NeuroMab Facility; Kirmiz et al., 2018b), and mouse IgG1 monoclonal anti-ryanodine receptor antibody (1 μg/ml, clone 34C, Thermo Fisher Scientific; this antibody detects all ryanodine receptor isoforms in mouse tissue; King et al., 2014; Yang et al., 2016; Irie and Trussell, 2017). The specificity of the anti-Kv2.1 antibody was validated by immunoblotting against wild-type and Kv2.1-KO mouse brain samples (datasheet). The anti-Kv2.2 antibody was validated by immunofluorescence labeling of wild-type and Kv2.2-KO mouse brain slices (datasheet). A detailed description of the primary antibodies is provided in Table 1. Furthermore, slices were washed with PBS-X, and then incubated for 4 h at room temperature in the incubation buffer containing the following secondary antibodies at a 1:500 dilution: CF405S goat anti-mouse IgG2a (20 381, Biotium), Alexa Fluor (AF) 488 goat anti-mouse IgG3 (A21151, Thermo Fisher Scientific), CF568 goat anti-mouse IgG2b (20 268, Biotium), and AF 647 goat anti-mouse IgG1 (A21240, Thermo Fisher Scientific). The slices were then washed with PBS-X, and finally coverslipped with Fluoromount-G (Southern Biotech).

**Table 1:** Antibody information

Antigen and antibody name	Immunogen	Species/isotype	Manufacture information	Concentration used
Ankyrin-G (N160/36)	Fusion protein ∼1000 aa of Ankyrin-G	Mouse IgG2a monoclonal antibody (mAb)	NeuroMab catalogue 75–146-020, RRID: AB_10673030	1 μg/ml
Kv2.1 (L80/21)	Synthetic peptide 837–853 aa of rat Kv2.1	Mouse IgG3 mAb	NeuroMab catalogue 75-315, RRID: AB_2315863	1 μg/ml
Kv2.2 (N372B/60)	Fusion protein 717–907 aa of rat Kv2.2 long isoform	Mouse IgG2b mAb	NeuroMab catalogue 75-360, RRID: AB_2315868	1 μg/ml
Ryanodine receptor (34C)	Partially purified chicken pectoral muscle ryanodine receptor	Mouse IgG1 mAb	Thermo Fisher Scientific catalogue MA3-925, RRID: AB_2254138	1 μg/ml

Intracellular labeling combined with fluorescence immunohistochemistry was performed as follows: cartwheel cells whose cell bodies exist in the surface of the acute brain slices (200-μm thickness) were whole cell patch-clamped with the pipette containing 0.5% (w/v) biocytin (Sigma-Aldrich) for >10 min. Slices were then fixed in 4% formaldehyde for 30 min at room temperature. Slices were washed three times with PBS-X and incubated with mouse IgG3 monoclonal anti-Kv2.1 antibody, and mouse IgG2b monoclonal anti-Kv2.2 antibody overnight at 4°C. Slices were washed with PBS-X, and then incubated for 4 h at room temperature in the incubation buffer containing AF 488 goat anti-mouse IgG3, CF568 goat anti-mouse IgG2b, and AF 647 streptavidin (4 μg/ml, S21374, Thermo Fisher Scientific). The slices were then washed, and coverslipped. Considering that the thickness of the slices, images of the immunoreactivities were taken from the surface of the slices (within 10 μm in depth).

Immunofluorescence images were acquired using a confocal microscope (A1R, Nikon) with the following appropriate settings: CF405S (excitation, 405-nm laser; emission, 425- to 475-nm bandpass filter), AF 488 (excitation, 488 nm; emission, 500–550 nm), CF568 (excitation, 561 nm; emission, 570–620 nm), and AF 647 (excitation, 640 nm; emission, 662–737 nm). Images were obtained with a 60 ×/1.4 numerical aperture oil-immersion objective lens, and the confocal pinhole size was 1.0 airy unit. Then, Z-stack images of each dye were taken sequentially, and the image stacks were deconvoluted to remove out-of-focus signals with NIS Elements software (Nikon). The profiles of the signal intensities along cell membranes were measured by drawing 0.042 μm in width lines with the aid of the ryanodine signal, which overlaps with cell membranes of cartwheel cells (Irie and Trussell, 2017). Lastly, immunofluorescent data were analyzed using Fiji software (https://fiji.sc/).

### Acute brainstem slice preparation, electrophysiological recordings, and data analysis

Acute brainstem slices containing the DCN were prepared first by anesthetizing the mice deeply with isoflurane and decapitating them. Then, parasagittal slices of brain stems (200-μm thickness) were prepared using a micro slicer (PRO7, Dosaka) in ice-cold, cutting solution containing the following: 93 mm *N*-methyl-D-glucamine, 2.5 mm KCl, 1.2 mm NaH_2_PO_4_, 30 mm NaHCO_3_, 20 mm HEPES, 0.5 mm CaCl_2_, 10 mm MgSO_4_, 5 mm sodium L-ascorbate, 2 mm thiourea, 3 mm sodium pyruvate, 3 mm myo-inositol, and 25 mm glucose, pH adjusted to 7.4 with HCl, and bubbled with 5% CO_2_/95% O_2_. Slices were transferred to room temperature (23–24°C) artificial CSF (ACSF) solution containing the following: 125 mm NaCl, 2.1 mm KCl, 1.7 mm CaCl_2_, 1 mm MgCl_2_, 1.2 mm KH_2_PO_4_, 20 mm NaHCO_3_, 3 mm HEPES-Na, 10 mm glucose, 0.4 mm ascorbic acid, 3 mm myo-inositol, and 2 mm sodium pyruvate, and bubbled with 5% CO_2_/95%O_2_. The slices were then incubated for 40 min before use. In electrophysiological recordings, ascorbic acid was omitted from the ACSF. The ACSF was supplemented with fast synaptic blockers [10 μm NBQX (Alomone Labs), 5 μm MK-801, 1 μm strychnine, and 100 μm picrotoxin] unless otherwise stated. The brainstem slices were transferred to a recording chamber and continuously perfused at 3 ml/min with ACSF at 33–34°C using a peristaltic pump (Minipuls 3, Gilson) unless otherwise stated. Next, neurons were visualized with an upright microscope (BX51WI, Olympus) equipped with a 60 ×/1.0 numerical aperture water-immersion objective lens and near infrared-CCD camera (C3077-79; Hamamatsu Photonics). Cartwheel cells were identified based on their location within the DCN, somatic size and morphology, and characteristic responses to current injections (Golding and Oertel, 1997; Tzounopoulos et al., 2004; Kim and Trussell, 2007; Roberts et al., 2008). Lastly, data were collected with Molecular Devices hardware and software (Multiclamp 700B, Digidata 1440A, and Clampex 10.3). Signals were low-pass filtered at 6 kHz and digitized at 20–100 kHz.

Whole-cell patch-clamp recordings were made using patch pipettes made from borosilicate glass capillaries (1B150F-4, WPI), which have a resistance of 2.5–3.5 ΜΩ when filled with a potassium gluconate-based internal solution containing the following: 125 mm K-gluconate, 10 mm KCl, 0.1 mm EGTA, 2 mm Mg-ATP, 3 mm Na_2_-ATP, 0.3 mm Na_2_-GTP, 13 mm Na_2_-phosphocreatine, and 10 mm HEPES. The pH was adjusted to 7.3 using KOH. In some experiments, 0.5% (w/v) biocytin was added to the pipette solution. In the current clamp recordings, series resistance was compensated using bridge balance, pipette capacitance was neutralized, and resting membrane potentials were adjusted to suppress spontaneous firing by injecting negative bias currents. When the voltage clamp recordings were made, series resistance was compensated by 60–80%, and the resistance was frequently monitored by turning off the compensation and applying 5-mV short step pulses (30-ms duration). If the series resistance changed by >10%, the records were rejected. Tail currents were measured at room temperature (23–24°C) using the following protocol: outward currents were evoked by voltage steps from –80-mV holding potential up to 30 mV in 10-mV increments for 50 ms, followed by repolarization to –50 mV for 250 ms. GxTX-sensitive tail current amplitudes were normalized to the maximal current, and normalized conductance (*G*) was plotted as a function of voltage. *G* was obtained by dividing the peak tail current by the electrochemical driving force: [*G* = *I*_K_/(*V* – *E*_K_)]. The activation curves (*G*/*G*_max_) were fitted with the Boltzmann function, *G*/*G*_max_ = 1/[1 + exp (*V*_1/2_ – *V*)/*k*], where *V*_1/2_ is the voltage at which 50% of the channels are activated, and *k* is a slope factor. The ACSF for outward current was further supplemented with 0.5 μm TTX (Alomone Labs), 100 nm apamin (a SK channel blocker, Alomone Labs), and 1 mm penitrem A (a BK channel blocker, Alomone Labs). To remove inward currents induced by voltage-gated calcium channels, CaCl_2_ in the ACSF was excluded and replaced with equimolar MgCl_2_, and 0.25 mm EGTA-Na (pH adjusted to 7.4 with NaOH) was added. Activation and deactivation of the GxTX-sensitive current were fit with a single exponential function to obtain decay time constants. Current clamp was used to assess the effects of GxTX on intrinsic membrane properties. As cartwheel cells exhibit spontaneous firing *in vitro* (Manis et al., 1994; Kim and Trussell, 2007; Bender et al., 2012), the resting membrane potential was adjusted to around −80 mV at the beginning of the experiment to suppress spontaneous firing by injecting negative bias current. The amplitude of the bias current was kept constant throughout the experiment. Input resistance was measured by applying a small hyperpolarizing current pulse (−50 pA, 300-ms duration). For the measurement of action potential properties, simple or complex spikes were induced by brief, strong current injection (1-ms duration, 100-pA increment, up to 2500 pA). The threshold current was defined as the current amplitude that evoked action potential for the first time, while the fast afterhyperpolarization (fAHP) was defined as the most negative voltage between first and second action potentials in complex spikes. The half-width of action potential was measured at the potential between the threshold and action potential peak. In complex-spiking neurons, the threshold potential, action potential amplitude, and the maximum rate of spike rise and decay of action potentials were calculated from the waveform of the first action potential. EPSCs were recorded with a CH_3_CsO_3_S-based internal solution containing the following: 87 mm CH_3_CsO_3_S, 20 mm CsCl, 5 mm CsF, 10 mm TEA, 0.1 mm EGTA, 2 mm Mg-ATP, 3 mm Na_2_-ATP, 0.3 mm Na_2_-GTP, 13 mm Na_2_-phosphocreatine, 2 mm QX-314 Cl (Alomone Labs), and 10 mm HEPES. The pH of the solution was adjusted to 7.3 using CsOH. Moreover, EPSCs were recorded in the presence of 1 μm strychnine and 100 μm picrotoxin, and the membrane potentials were held at −80 mV under the voltage clamp configuration. EPSCs were evoked by electrical stimulation of parallel fibers with an ACSF-filled patch pipette (Tzounopoulos et al., 2004; Roberts and Trussell, 2010). The stimulus electrode was driven by the combination of an isolator (SS-203J, Nihon Kohden) and an electronic stimulator (SEN-7203, Nihon Kohden). The stimulus intensity was 34–89 V (100-μs duration). A train of EPSCs was evoked, and averages of five traces were used for the analysis. Synaptically evoked action potentials were also induced by electrical stimulation of parallel fibers. We defined that successful action potential has maximum rate of rise >30 V/s and peak amplitude higher than −15 mV. This is because in cartwheel cells, depolarization having 30-V/s maximum rate of rise elicits IPSCs at ∼50% failure rate (Roberts et al., 2008). Moreover, in the cells, action potentials which have peak amplitude lower than approximately −15 mV cannot trigger transmitter release (Roberts et al., 2008). Maximum rate of rise was obtained by differentiating voltage response evoked by parallel fibers stimulation.

Data were analyzed using Clampfit 10.3 software (Molecular Devices) and Igor Pro 6 software (Wavemetrics) with the added import functionality provided by ReadPclamp XOP of the NeuroMatic software package (http://www.neuromatic.thinkrandom.com/; Rothman and Silver, 2018). Liquid junction potentials (K-gluconate-based, −10 mV; CsCl-based, −5 mV) were corrected offline. All data are provided as mean ± SEM unless otherwise stated. Numbers in parentheses in figures and *n* in the text and tables indicate the number of replications (cells). Statistical significance was tested using paired *t* tests unless otherwise stated (significance, *p *<* *0.05). GraphPad Prism 5 (GraphPad Software) was used for the statistical analysis.

### Peptide blocker application

When the peptide blocker GxTX (Alomone Labs) was used, 0.1 mg/ml bovine serum albumin was added to all ACSF to reduce non-specific binding. The final concentration of GxTX was 100 nm. GxTX was perfused for at least 5 min through recirculation with a peristaltic pump (Minipuls 3) before data recording.

## Results

### Subcellular localization of Kv2.1 and Kv2.2 channels in cartwheel cells

**Figure 1.**
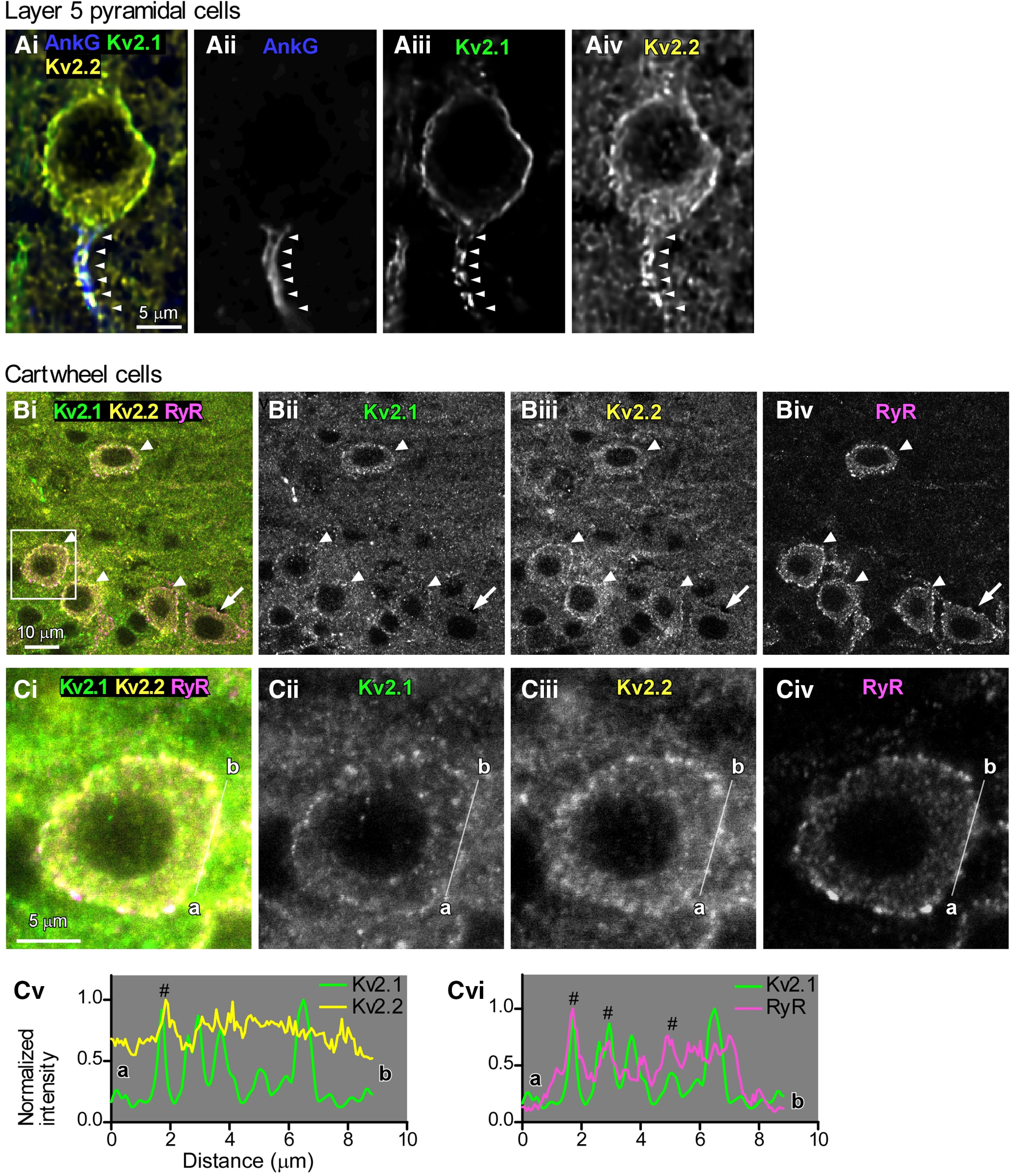
Immunoreactivity of Kv2.1 and Kv2.2 on somatic membrane of cartwheel cells. ***A***, Single optical section of a layer 5 cortical neuron immunolabeled for ankyrin-G (AnkG; blue), Kv2.1 (green), and Kv2.2 (yellow). Ankyrin-G-positive AIS is indicated by arrowheads. ***Bi–Biv***, Single optical section of cartwheel cells immunolabeled for Kv2.1 (green), Kv2.2 (yellow), and ryanodine receptors (RyR; magenta). The cell bodies of cartwheel cells are pointed by arrowheads or arrows. Note that one cartwheel cell is Kv2.1-negative (arrows). ***Ci–Civ***, Zoom-in image from the rectangle area in ***Bi***. ***Cv***, ***Cvi***, The profiles of signal intensity along the membrane measured by drawing linear regions of interest along the cell membrane (a − b in ***Ci–Civ***), guided by the ryanodine receptor signal. Overlapped peaks are indicated by hash marks (#).

First, to verify the reproducibility of fluorescence immunolabeling using anti-Kv2.1 and anti-Kv2.2 antibodies, layer 5 pyramidal cells of mouse cerebral cortex were labeled, as shown in Figure 1*A*. Consistent with a previous report (Kirmiz et al., 2018a), both Kv2.1 and Kv2.2 signals were observed along the somatic membrane. Moreover, Kv2.1 and Kv2.2 signals were also detected in the ankyrin-G-positive AIS (Fig. 1*A*, arrowheads).

**Figure 2.**
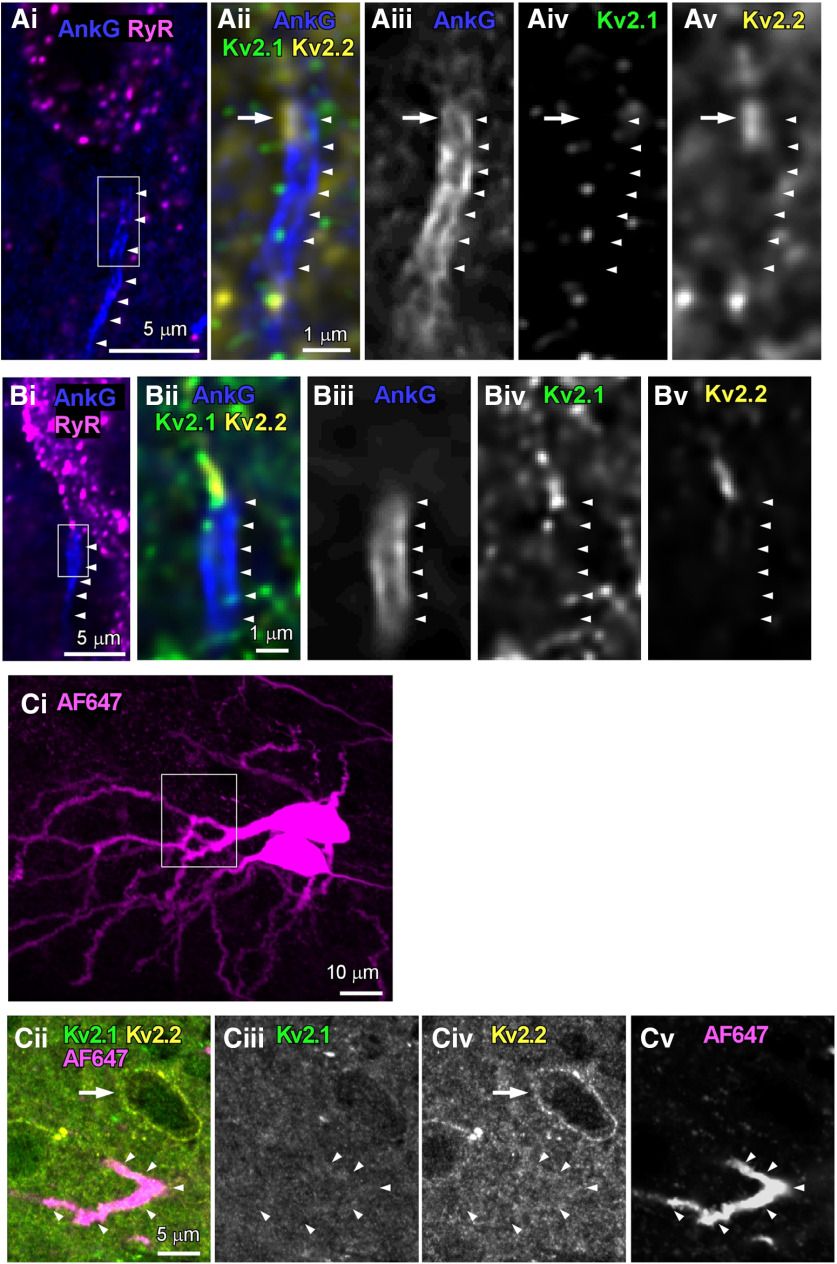
Immunoreactivity of Kv2.1 and Kv2.2 on ankyrin-G-positive AISs and dendrites of cartwheel cells. ***Ai***, ***Bi***, Projected z-stack of optical sections taken from cartwheel cells immunolabeled for ryanodine receptors (RyR; magenta) and ankyrin-G (AnkG; blue). ***Aii–Av***, ***Bii–Bv***, Single optical sections of AISs immunolabeled for ankyrin-G (AnkG; blue), Kv2.1 (green), and Kv2.2 (yellow). The positions of AISs are indicated by arrowheads. The location of Kv2.2 on the edge of AIS is pointed by arrows in ***Aii–Av***. The cell in ***A*** is distinct from the one in ***B***. ***Ci***, Projected z-stack of optical sections of AF 647-labeled cartwheel cells. Two cartwheel cells are labeled. ***Cii–Cv***, Single optical sections of a proximal dendrite, which is immunonegative to Kv2.1 (***Cii***) and Kv2.2 (***Civ***). Location of the dendrite was marked by arrowheads. Note that a cell is Kv2.2-positive in the panels (arrow), indicating that the antibodies used reached the tissue.

Kv2.1 clusters are juxtaposed to clustered ryanodine receptors in specific neurons, including CA1 pyramidal neurons and striatal medium spiny neurons (Mandikian et al., 2014). To examine whether Kv2.1 clusters in cartwheel cells overlapped with ryanodine receptors and/or Kv2.2, cartwheel cells were immunolabeled with anti-Kv2.1, anti-Kv2.2, anti-ryanodine receptor, and anti-ankyrin-G antibodies (Figs. 1*B*,*C*, 2*A*,*B*). Figure 1*B* illustrates the immunofluorescence labeling of Kv2.1, Kv2.2, and ryanodine receptors. Cartwheel cells were easily identified in the DCN with the aid of the puncta of the ryanodine receptor along the cell membrane (Fig. 1*Biv*; Yang et al., 2016; Irie and Trussell, 2017). Puncta of Kv2.2 signals were observed in cartwheel cells (Fig. 1*Biii*, arrowheads and arrows). On the other hand, those of Kv2.1 were detected most of cartwheel cells (Fig. 1*Bii*, arrowheads), but some of them were immunonegative to Kv2.1 (Fig. 1*Bii*, arrows). We measured the immunopositive rate of Kv2.1 and Kv2.2 in ryanodine-positive cartwheel cells (Table 2). Indeed, ∼25% of cartwheel cells’ bodies did not show clear Kv.2.1 immunoreactivity, but almost all of cartwheel cells’ bodies were Kv2.2-positive. The profiles of signal intensity along the cell body’s membrane were measured by drawing linear regions of interest along the cell membrane, guided by the ryanodine receptor signal (Fig. 1 *Ci–Civ*). The signal intensities of Kv2.1 and Kv2.2, or those of Kv2.1 and ryanodine receptors along the linear regions of interest are plotted in Figure 1*Cv*,*Cvi*, respectively. In Figure 1*Cv*, the profile had one peak that overlapped between the Kv2.1 and Kv2.2 signals (a hash mark in Fig. 1*Cv*). In Figure 1*Cvi*, the profile had three peaks that overlapped between the Kv2.1 and ryanodine receptor signals (hash marks in Fig. 1*Cvi*). We also labeled hippocampal slices with anti-Kv2.1 and anti-ryanodine receptor antibodies as the positive control, and confirmed that Kv2.1 clusters were juxtaposed to the puncta of ryanodine receptors on the membrane of the cell bodies’ of CA1 pyramidal neurons (data not shown), as reported previously (Kirmiz et al., 2018a). These results indicate that cartwheel cell bodies express Kv2.2 and most of them also have Kv2.1, and that the puncta of Kv2.1 do not overlap completely with those of Kv2.2 or that of ryanodine receptors.

**Table 2:** Quantitative analysis of Kv2.1 and Kv2.2 immunoreactivities in cartwheel cells

	Cell bodies in p27 male	Cell bodies in p30 male	Cell bodies in p34 female	Cell bodies in the three mice	AISs in the three mice	Proximal dendrites in biocytin-filled cells
Kv2.1(+) and Kv2.2 (+)/total	25/37 (67.6%)	31/43 (72.1%)	36/50 (72.0%)	92/130 (70.8%)	0/12 (0.0%)	0/10 (0.0%)
Kv2.1(−) and Kv2.2 (+)/total	11/37 (29.7%)	10/43 (23.3%)	13/50 (26.0%)	34/130 (26.2%)	0/12 (0.0%)	0/10 (0.0%)
Kv2.1(+) and Kv2.2 (-)/total	0/37 (0%)	0/43 (0%)	0/50 (0%)	0/130 (0%)	0/12 (0.0%)	0/10 (0.0%)
Kv2.1(−) and Kv2.2 (−)/total	1/37 (2.7%)	2/43 (4.7%)	1/50 (2.0%)	4/130 (3.1%)	12/12 (100.0%)	10/10 (100.0%)

For the examination of immunoreactivity to cell bodies and AISs, ryanodine-positive, medium-sized cells were considered as cartwheel cells. When cartwheel cells were labeled intracellularly, the cell type was confirmed by recording the characteristic complex spiking behavior.

Using multiple immunofluorescence labeling, we explored the presence of Kv2.1 and Kv2.2 signals on AIS, which is a specialized membrane region in the axons of neurons where action potentials are initiated (Kole and Stuart, 2012). Because AISs express the cytoskeletal scaffolding protein ankyrin-G (Kole and Stuart, 2012), AISs were visualized by labeling ankyrin-G with anti-ankyrin-G antibody (Fig. 2*A*,*B*, arrowheads). Cell bodies of cartwheel cells were labeled with anti-ryanodine receptor antibody. In Figure 2*A*, the ankyrin-G signal slightly overlapped with Kv2.2 (Fig. 2*Aii–Av*, arrow), but not with Kv2.1. Another AIS is shown in Figure 2*B*, and no overlap was observed between ankyrin-G signal and Kv2.1 or Kv2.2. We quantify the immunolabeling in AISs in Table 2, showing that none out of 12 AISs exhibited the presence of Kv2.1 and/or Kv2.2 signals.

In addition to cell bodies and AISs, the localization of Kv2.1 and Kv2.2 were explored in proximal dendrites of cartwheel cells (Fig. 2*C*). Cell bodies and dendrites were labeled using intracellular infusion of biocytin via patch pipette, and the dendrites were visualized by using streptavidin-conjugated AF 647 (Fig. 2*Ci*,*Cv*). Figure 2*Cii–Cv* show immunoreactivity of Kv2.1 and Kv2.2 in proximal dendrite, indicating the absence of Kv2.1 or Kv2.2 labeling. The immunoreactivities in dendrites were summarized in Table 2, demonstrating that none out of 10 proximal dendrites showed immunopositive to Kv2.1 and/or Kv2.2. However, because Kv2.1 and Kv2.1 immunoreactivity in distal dendrites could not be evaluated, significant Kv2 expression may exist there (see Discussion). Taken together, these results suggest that AISs and proximal dendrites in cartwheel cells lack Kv2 channels, and cartwheel cell bodies largely express both Kv2.1 and Kv2.2 on the somatic membrane.

### Biophysical property of Kv2 current in cartwheel cells

**Figure 3.**
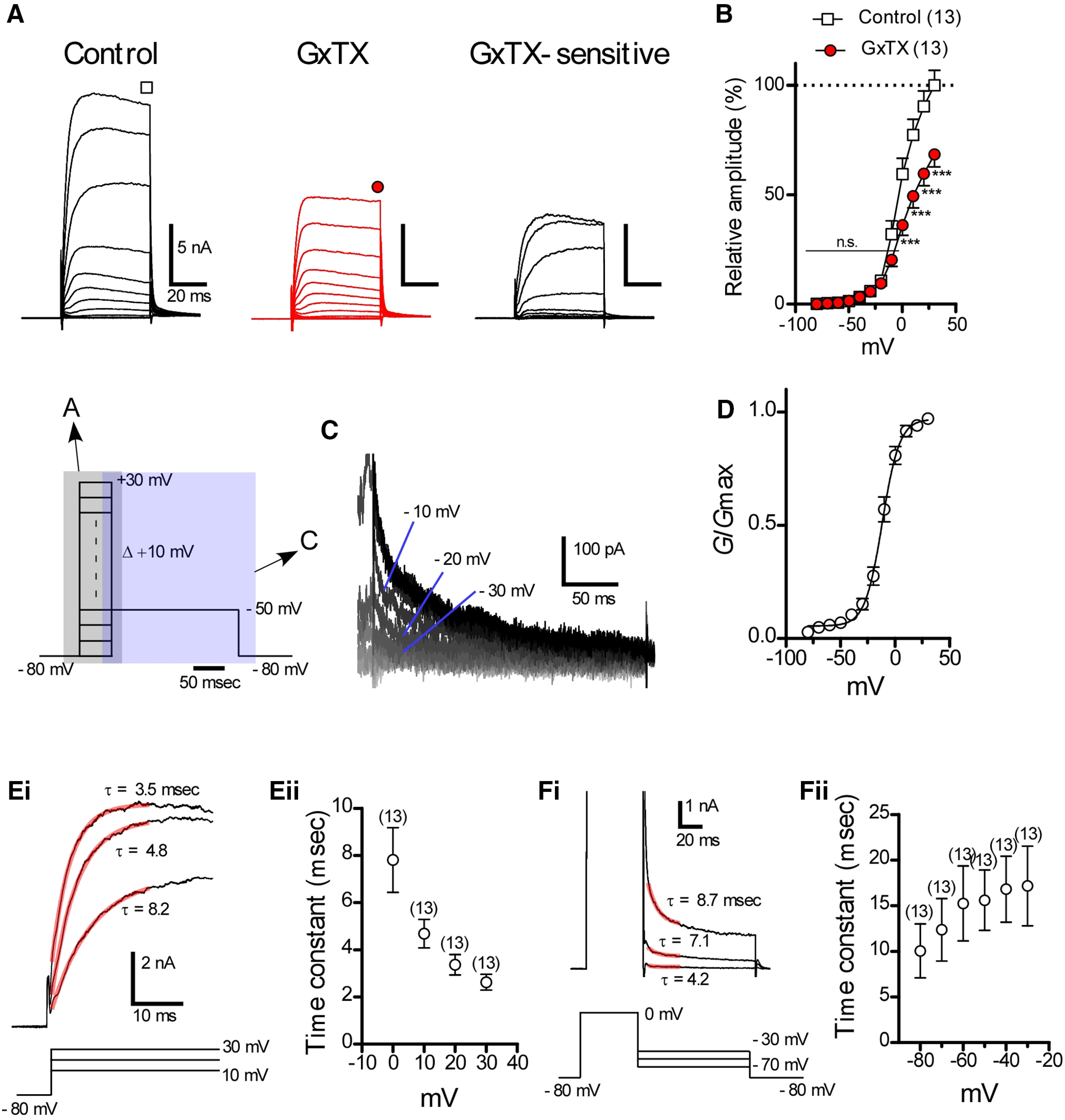
Biophysical properties of GxTX-sensitive Kv2 current in cartwheel cells. ***A***, Outward current evoked by voltage steps. Pulse protocol is indicated at the bottom of ***A***. The recordings were made at room temperature (23–24°C) using ACSF supplemented with NBQX, MK-801, strychnine, picrotoxin, TTX, apamin (a SK channel blocker), and penitrem A (a BK channel blocker). To remove inward current by voltage-gated calcium channels, CaCl_2_ in the ACSF was excluded and replaced with equimolar MgCl_2_, and 0.25 mm EGTA-Na was added. GxTX-sensitive current was obtained by subtraction. ***B***, The current–voltage relationship of the outward current in the absence (control) or presence of 100 nm GxTX (GxTX). The amplitude of steady-state was used for the plotting. Here and the following figures, error bars indicate SEM, numbers in parentheses indicate the number of replications (cells). Statistical significance was tested using two-way repeated measure ANOVA and Bonferroni *post hoc* tests (significance at *p *<* *0.05). n.s.: not significant; ***p < 0.001. ***C***, GxTX-sensitive tail current evoked by voltage steps. The voltage pulse protocol is shown in the left of ***C***. ***D***, GxTX-sensitive, normalized conductance (*G*) plotted as a function of voltage (*n* = 13 in each point). The activation curves were fit with the Boltzmann function with a *V_1/2_* of –11.84 ± 0.86 mV (*n* = 13) and a slope factor (*k*) of 7.90 ± 0.76 mV (*n* = 13). ***Ei***, Activation kinetics of Kv2 current isolated by GxTX subtraction as in ***A***. Activation was fit with a single exponential function, and τ is the time constant. Fits are shown as red lines. ***Eii***, τ as a function of voltage. ***Fi***, Deactivation kinetics of Kv2 current obtained by GxTX subtraction. Deactivation was fit with a single exponential function, and fits are shown as red lines. ***Fii***, τ as a function of voltage.

After confirming the presence of Kv2 channels in cartwheel cells by immunofluorescence labeling, we recorded GxTX-sensitive Kv2 current using the whole-cell patch-clamp method and GxTX, a specific Kv2 channel blocker (Herrington et al., 2006; Herrington, 2007). The recordings were performed at room temperature (23–24°C) to reduce the current amplitude, and 100 nm GxTX-containing ACSF was perfused for longer than 5 min. The ACSF was supplemented with fast synaptic blockers, 0.5 μm TTX, 100 nm apamin, and 1 mm penitrem A. To remove the current from voltage-gated calcium channels, CaCl_2_ in the ACSF was replaced with equimolar MgCl_2_, and 0.25 mm EGTA-Na was added. Figure 3*A* shows the outward current in the absence or presence of GxTX recorded from the same cell and GxTX-sensitive current obtained by subtraction. Outward current was induced by depolarizing pulses up to 30 mV, followed by repolarization to –50 mV. The detailed voltage pulse protocol used is shown at the bottom of Figure 3*A*. In the absence of GxTX (Fig. 3*A*, control), the detectable outward current was first activated at voltage –40 mV, and grew with further depolarization, exhibiting a small amount of inactivation. Bath application of GxTX dramatically reduced the outward current (Fig. 3*A*, GxTX). The current–voltage relationships of outward current recorded under control conditions (Fig. 3*A*, control) and of that obtained in the presence of GxTX (Fig. 3*A*, GxTX) are plotted in Figure 3*C*. The current amplitude was measured at the end of depolarization pulses and then normalized to the average amplitude of the control at 30 mV. Outward current was significantly reduced by GxTX at 0–30 mV (**p <* *0.01, ***p <* *0.001 by two-way repeated measure ANOVA and Bonferroni *post hoc* tests, *n* = 13). To define the voltage dependence of steady-state activation, an activation curve was constructed from averaged data in 13 cartwheel cells using tail current after 50-ms depolarization pulses (Fig. 3*C*,*D*). The experimental data were fit well by a Boltzmann curve (Fig. 3*D*, black curve), with a *V_1/2_* of –11.84 ± 0.86 mV (*n* = 13) and a *k* of 7.90 ± 0.76 mV (*n* = 13). This *V_1/2_* value is very close to *V_1/2_* that was recorded in superior cervical ganglion neurons (*V_1/2_* = –12.9) and in entorhinal cortex layer II stellate cells (*V_1/2_* = –13.4), suggesting that the voltage clamp experiment was conducted accurately (Liu and Bean, 2014; Hönigsperger et al., 2017).

Figure 3*E* illustrates the activation kinetics of the Kv2 current obtained by GxTX subtraction. The rising phase (20 ms starting from the depolarization onset) of Kv2 current was fitted by a single exponential function (Fig. 3*Ei*, red curves). Figure 3*Eii* summarizes the voltage dependence of the activation time constant. The value became smaller as the membrane potential became more depolarized. Figure 3*F* shows the deactivation kinetics of the Kv2 current obtained by GxTX subtraction. The deactivation phase (5–35 ms from the repolarization onset) was also fitted by a single exponential function (Fig. 3*Fi*, red curves). The time constant tended to increase as the repolarization became positive (Fig. 3*Fii*). Thus, Kv2 current in cartwheel cells is high-voltage activated and activates and deactivates slowly (Johnston et al., 2010).

### The blockade of Kv2 channels depolarizes the potential of afterdepolarization (ADP)

**Figure 4.**
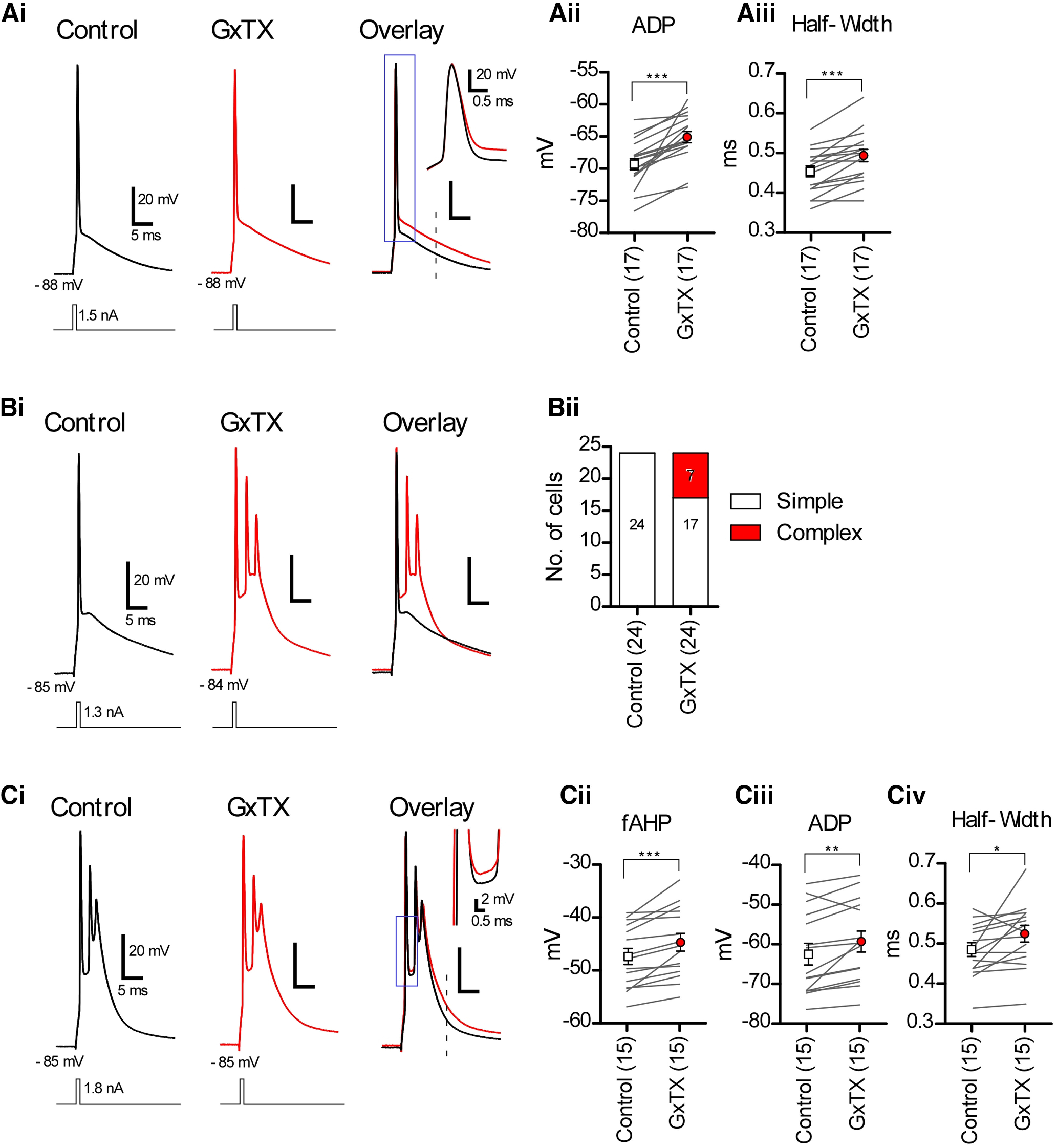
The effects of GxTX on simple or complex spikes elicited by brief current injection. ***Ai***, Simple spike evoked by current injection (1-ms duration). Recordings were made in the ACSF at 33–34°C supplemented with NBQX, MK-801, strychnine, and picrotoxin. GxTX broadened the action potential width (inset) and depolarized the potential of ADP. ***Aii***, Summary of the change ADP measured at 10 ms after the beginning of depolarizing current injection (dashed line in ***Ai***, overlay). ***p < 0.001. ***Aii***, Summary of the half-width of action potentials; ***p < 0.001. ***Bi***, The simple spike converted into complex spikes by GxTX. ***Bii***, Summary of the change of spiking pattern by GxTX. Out of 24 simple-spiking cells, seven cells started showing complex spiking in GxTX. ***Ci***, Complex spikes evoked by current injection. In GxTX, fAHP became less negative (inset) and ADP became more prominent. ***Cii–Civ***, Summary of fAHP, ADP, and half-width of first action potential in complex spikes. ADP was measured at 10 ms after the beginning of depolarizing current injection (dashed line in ***Ci***, overlay). Statistical significance was tested using paired *t* tests; *p < 0.05, **p < 0.01, ***p < 0.001.

In cartwheel cells, *V_1/2_* of Kv2 current was approximately –11.8 mV; therefore, Kv2 channels should be activated during action potentials. To confirm this hypothesis, we performed whole-cell current clamp recording from cartwheel cells at near-physiological temperature (33–34°C). As cartwheel cells exhibit spontaneous firing *in vitro* (Manis et al., 1994; Kim and Trussell, 2007; Bender et al., 2012), resting membrane potentials were adjusted to suppress spontaneous firing by injecting negative bias currents (–50 to –150 pA). Cartwheel cells show two types of action potentials: simple spikes and complex spikes (Manis et al., 1994; Kim and Trussell, 2007). When a brief (1-ms duration) depolarizing current was injected to cartwheel cells, we observed either simple spikes (Fig. 4*Ai*,*Bi*, control) or complex ones (Fig. 4*Ci*, control). Here, we termed cells exhibiting simple spikes as simple-spiking cells, and complex ones as complex-spiking cells. Out of 39 cells, 24 corresponded to simple-spiking cells, and the rest were complex-spiking (15 cells). In simple-spiking cells, we observed three effects of GxTX on action potential behavior: depolarization of the ADP (Fig. 4*A*), broadening of the action potential (Fig. 4*A*), and conversion of simple-spiking to complex-spiking (Fig. 4*B*). The magnitude of the ADP was measured 10 ms after the beginning of depolarizing current injection (Fig. 4*Ai*, overlay, dashed line), as summarized in Figure 4*Aii*, and revealed that it was depolarized significantly by 4.25 mV in the presence of GxTX (–69.23 ± 0.88 mV, GxTX, –65.01 ± 0.90 mV, *n* = 17, ***p < 0.001; Fig. 4*Aii*, control). The half-width of the action potential was also significantly broadened by GxTX (0.452 ± 0.013 ms, GxTX, 0.492 ± 0.015 mV, *n* = 17, ***p < 0.001; Fig. 4*Aiii*, control). Out of 24 simple-spiking cells, 7 cells started showing complex spikes when Kv2 channels were blocked by GxTX (Fig. 4*Bi*,*Bii*). Presumably this change was because of the more depolarized ADP in GxTX which reached the threshold of the action potential, thus driving extra spikes. When complex-spiking cells were exposed to GxTX-containing ACSF, the fAHP between the first and second spikes became less negative (Fig. 4*Ci*, overlay, inset; –47.51 ± 1.52 mV, GxTX, –44.85 ± 1.67 mV, *n* = 15, ***p < 0.001; Fig. 4*Cii*, control). Moreover, the potential of ADP recorded after complex spikes became positive (Fig. 4*Ci*, overlay). The potential was measured at 10 ms after the beginning of depolarizing current injection (Fig. 3*Ci*, overlay, dashed line), and the mean of the shift was 3.13 mV (–62.51 ± 2.70 mV, GxTX, –59.38 ± 2.62 mV, *n* = 15, **p < 0.01; Fig. 4*Ciii*, control). In addition, the effects of GxTX on intrinsic membrane properties were also examined (Table 3). The maximum rate of spike decay was obviously slowed, and threshold current was significantly increased by GxTX. The other intrinsic membrane properties, including resting membrane potential, input resistance, threshold potential, action potential amplitude, maximum rate of spike rise, were not affected by GxTX (Table 3). These findings demonstrate that Kv2 channels lower the membrane potential of ADP in both simple-spiking and complex-spiking cells and accelerate the repolarization of an action potential.

**Table 3:** The effects of GxTX on intrinsic membrane properties of cartwheel cells

	Resting membrane potential (mV)		Input resistance (MΩ)		Threshold potential (mV)		Threshold current (pA)		Action potential amplitude (mV)		Maximum rate of spike rise (V/s)		Maximum rate of spike decay (V/s)	
	Control	GxTX	Control	GxTX	Control	GxTX	Control	GxTX	Control	GxTX	Control	GxTX	Control	GxTX
Simple-spiking (24)	–83.39 ± 0.62	−83.04 ± 0.53 *^n^*^.^*^s^*	77.03 ± 4.18	78.70 ± 3.71*^n^*^.^*^s^*	–62.49 ± 1.45	–61.27 ± 1.41 *^n^*^.^*^s^*	1808 ± 71	1704 ± 79**	75.90 ± 2.22	74.89 ± 2.61 *^n^*^.^*^s^*	567.9 ± 23.1	556.1 ± 23.9 *^n^*^.^*^s^*	–170.0 ± 8.25	–132.9 ± 5.34***
Complex-spiking (15)	−81.05 ± 0.69	–80.84 ± 0.65 *^n^*^.^*^s^*	89.56 ± 8.73	94.10 ± 8.20*^n^*^.^*^s^*^.^	–62.21 ± 1.39	–60.22 ± 1.69 *^n^*^.^*^s^*	1953 ± 72	1760 ± 83***	77.11 ± 2.02	74.72 ± 3.02 *^n^*^.^*^s^*	569.5 ± 26.4	560.9 ± 25.1 *^n^*^.^*^s^*	–141.5 ± 6.30	–129.9 ± 6.55*
Combined (39)	–82.49 ± 0.50	–82.20 ± 0.44 *^n^*^.^*^s^*	81.85 ± 4.28	84.62 ± 4.02 *^n^*^.^*^s^*	–62.38 ± 1.03	–60.87 ± 10.8 *^n^*^.^*^s^*	1864 ± 52	1726 ± 57***	76.37 ± 1.55	74.82 ± 1.96 *^n^*^.^*^s^*	568.5 ± 17.2	557.9 ± 17.4 *^n^*^.^*^s^*	–159.0 ± 6.00	–131.8 ± 4.09***

As cartwheel cells exhibit spontaneous firing *in vitro*, at the beginning of the experiment, the resting membrane potential was adjusted to around −80 mV to suppress spontaneous firing by injecting a negative bias current (−50 to −150 pA). The amplitude of the bias current was kept constant throughout the experiment. Input resistance was measured by applying a small hyperpolarizing current pulse (−50 pA, 300-ms duration). For the measurement of action potential properties, simple or complex spikes were induced by brief, strong current injection (1-ms duration, 100-pA increment, up to 2500 pA). Threshold current was defined as the current amplitude that evoked action potential for the first time. The half-width of action potential was measured at the potential between the threshold and action potential peak. In complex-spiking neurons, the threshold potential, action potential amplitude, and the maximum rate of spike rise and decay of action potentials were calculated from the waveform of the first action potential. Statistical significance was tested using paired *t* tests; n.s.: not significant, *p < 0.05, **p < 0.01, ***p < 0.001.

### Sustained firing induced by depolarizing current injection are interrupted by the blockade of Kv2 channels

**Figure 5.**
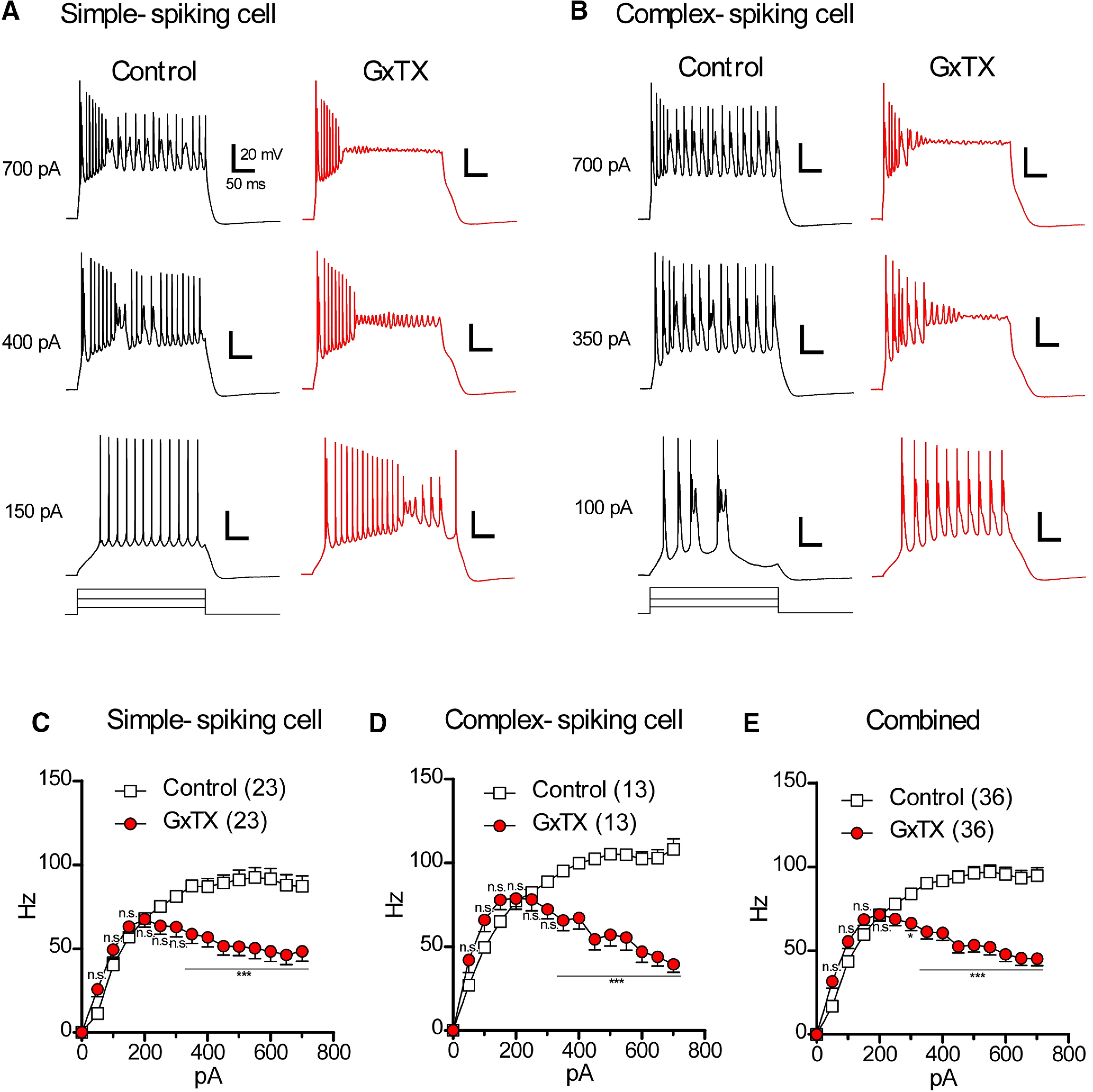
The effects of GxTX on sustained firing induced by depolarizing current injection. ***A***, The effects of GxTX on a simple-spiking cell. Tonic firing induced by square-wave current injection in the absence (control) or presence (GxTX) of GxTX. In GxTX, the cell showed phasic response when the cells were excited by 400- or 700-pA current injection. ***B***, The effects of GxTX on a complex spiking cell. The generation of trains of complex spikes disappear in the middle of current injection (GxTX, 350 and 700 pA). ***C–E***, Summary of the input-output relationship of simple-spiking cells (***C***), complex-spiking cells (***D***), and both simple-spiking and complex-spiking cells (***E***, combined). Statistical significance was tested using two-way repeated measure ANOVA and Bonferroni *post hoc* tests. n.s.: not significant; *p < 0.05, ***p < 0.001.

Figure 4 and Table 3 illustrate that Kv2 channels contribute to lower the potential of ADP and accelerate the falling phase of an action potential. However, because of their slow activation kinetics, Kv2 channels may not fully open during the time course of a single action potential. To determine what will happen if Kv2 channels are blocked during sustained depolarization in cartwheel cells, sustained firing was evoked by injecting a long (300-ms duration) square-wave depolarizing current (Fig. 5). Simple-spiking cells exhibited sustained firing characterized by simple spikes when weak current was injected (Fig. 5*A*, 150 pA). With stronger current injection, these cells could fire both simple and complex spikes (Fig. 5*A*, 400 and 700 pA), consistent with previous reports (Kim and Trussell, 2007). In the presence of GxTX, cells often stopped firing altogether in the middle of a sustained current step (Fig. 5*A*, GxTX, 400, and 700 pA). Figure 5*C* summarizes the input-output relationship of simple-spiking cells. The firing frequency was reduced by GxTX between 250- and 700-pA current injection. A similar tendency was observed in complex-spiking cells (Fig. 5*B*,*D*). In the control, depolarizing pulses produced either isolated complex spikes (Fig. 5*B*, control, 100 pA) or trains of complex spikes (Fig. 5*B*, control, 350, and 700 pA; Manis et al., 1994). In GxTX, weak current injection tended to induce more action potentials (Fig. 5*B*, GxTX, 100 pA), but the increase was not significant (Fig. 5*D*, GxTX, 50–150 pA, n.s.: not significant by two-way repeated measure ANOVA and Bonferroni *post hoc* tests). During stronger current injection, the cell ceased showing trains of complex spikes (Fig. 5*B*, GxTX, 350, and 700 pA). Figure 5*D*,*E* summarize the input-output relationship of complex-spiking cells and the combined data from both simple and complex-spiking cells. In both panels, the firing frequencies of the control data are seen to increase monotonically before reaching a plateau. On the other hand, the firing frequencies in GxTX increased in the range of 50–200 pA and then decreased as the injected current became stronger (*p < 0.05 and ***p < 0.001 by two-way repeated measure ANOVA and Bonferroni *post hoc* tests). These results clearly demonstrate that Kv2 channels are necessary not only for sustained tonic firing induced by current injection but also for preventing depolarization block in cartwheel cells.

### The blockade of Kv2 channels induces failure of sustained firing evoked by parallel fiber stimulation in a frequency-dependent manner

**Figure 6.**
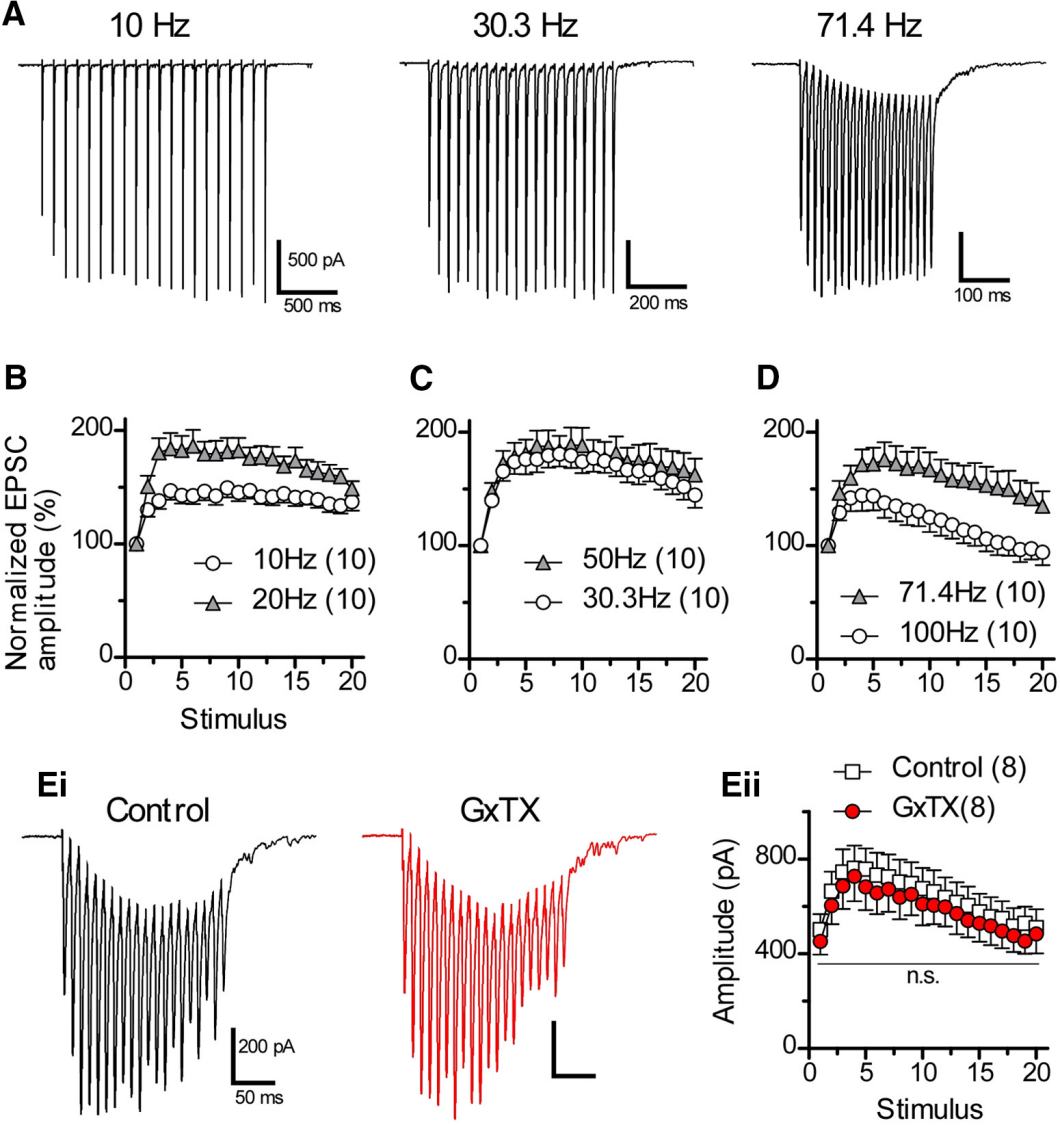
A train of parallel fiber-EPSCs evoked by various stimulus frequencies. ***A***, A train of EPSCs induced by repetitive stimulation of parallel fibers. The membrane potential was held at –80 mV in the presence of strychnine and picrotoxin. Averages of five traces were used. Note that the amplitude of the last EPSCs is still large compared with the first EPSC in every stimulus frequency. ***B–D***, Summary of relative amplitude of EPSCs plotted against stimulus numbers. ***Ei***, A train of EPSCs in the absence (control) or presence (GxTX) of GxTX. ***Eii***, Summary of the effects of GxTX on a train of EPSCs evoked at 100 Hz. Statistical significance was tested using two-way repeated measure ANOVA and Bonferroni *post hoc* tests. n.s.: not significant.

**Figure 7.**
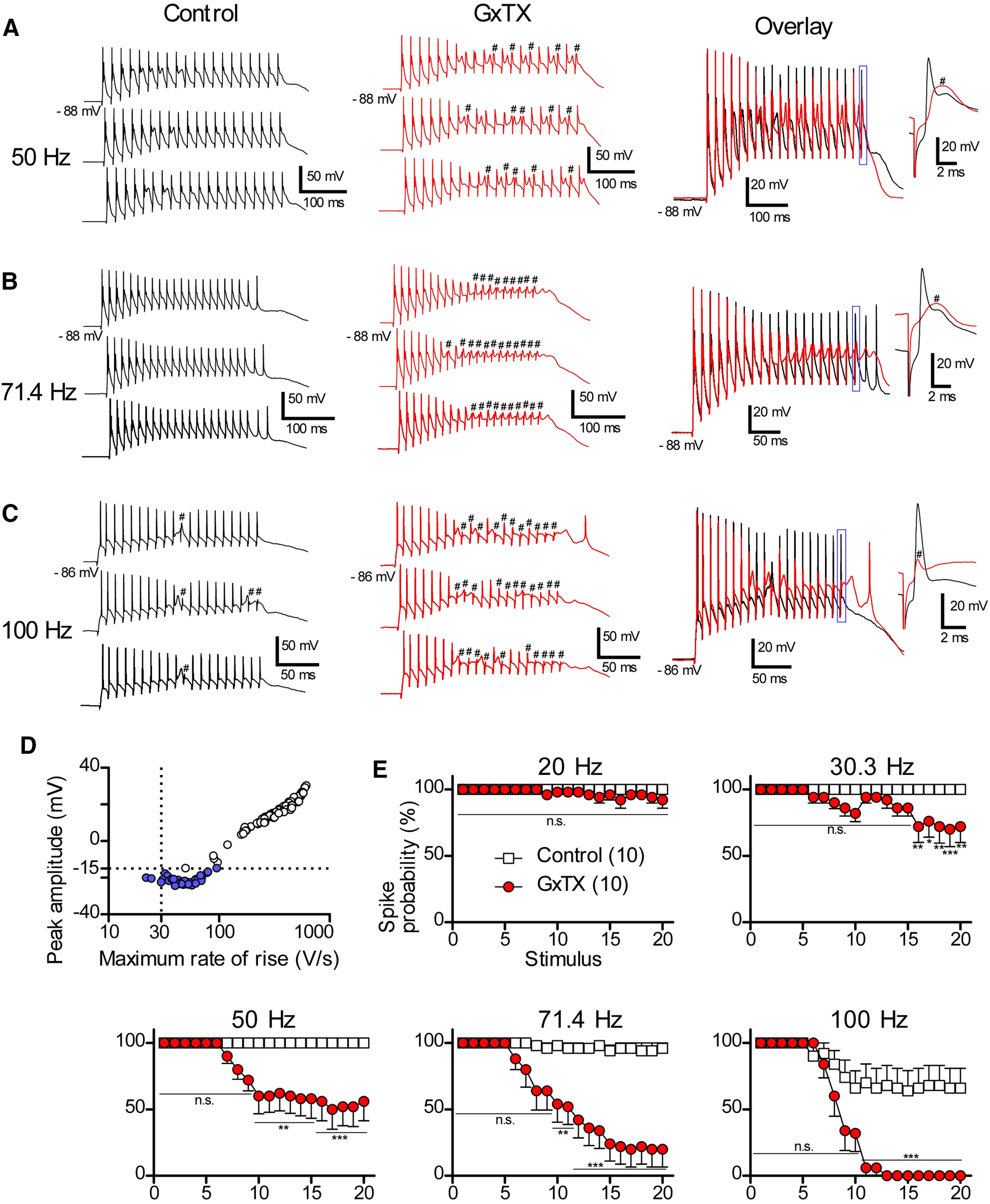
The effects of GxTX on sustained firing evoked by repetitive parallel fiber stimulation. ***A–C***, Tonic firing evoked by repetitive parallel fiber stimulation. Traces in ***A***, ***B*** were recorded from the same cells. Traces in ***C*** were from another cell. Top traces in each panel were used for the overlays. A part of overlayed traces is expanded as insets. Note that in the presence of GxTX (red traces), some of action potentials were not evoked by the stimulation. Failures of action potentials are indicated by hash marks (#). Stimulus artifacts were truncated for clarity. ***D***, The relationship between maximum rate of rise of spikes and peak amplitude of action potentials plotted on a semi-logarithmic scale. The plot was made by differentiating six traces showing 20 synaptic responses at 100 Hz in the presence or absence of GxTX. Action potentials which have the rate of rises lower than 30 V/s or have peak amplitude lower than –15 mV are colored in blue (failures). ***E***, Summary of spike probabilities plotted against stimulus numbers. For the analysis, five traces in control and five in GxTX were used from each recording for the calculation of the probabilities. Statistical significance was tested using two-way repeated measure ANOVA and Bonferroni *post hoc* tests. n.s.: not significant; *p < 0.05, **p < 0.01, ***p < 0.001.

The previous experiment explored sustained firing in response to a constant current injection. However, the responsiveness of the neurons might be quite different in response to trains of discrete EPSPs. To study the role of Kv2 channels under more physiological stimuli, we activated excitatory inputs to cartwheel cells. Cartwheel cell dendrites receive glutamatergic excitatory synaptic inputs from parallel fibers (Wouterlood and Mugnaini, 1984). Electrical stimulation of parallel fibers *in vitro* evokes EPSCs in cartwheel cells (Roberts and Trussell, 2010; Vogler et al., 2020), and activation of parallel fibers *in vivo* can drive action potentials in cartwheel cells (Davis and Young, 1997). Thus, parallel fiber input is considered to be the principal excitatory input in cartwheel cells (Wouterlood and Mugnaini, 1984). To examine how parallel fiber-induced action potentials are affected by the blockade of Kv2 channels, we first evaluated the effect of GxTX on parallel fiber-EPSC (Fig. 6), and then blocked Kv2 channels of cartwheel cells during repetitive action potentials (Fig. 7).

Cartwheel cells were held at –80 mV under voltage clamp conditions, and a train of stimuli (20 times) was applied on parallel fibers in the ACSF supplemented with 1 μm strychnine and 100 μm picrotoxin. The stimulus frequency was varied from 10 to 100 Hz. At 10 and 30.3 Hz, a train of EPSCs showed slight facilitation at the beginning of stimulation, and then sustained responses (Fig. 6*A*, 10 and 30.3 Hz). At 71.4 Hz, a train of EPSCs showed facilitation in the beginning, followed by a slight depression. Figure 6*B–D* summarize a series of experiments, revealing that parallel fiber-cartwheel cell synapses do not show clear synaptic depression, and that the amplitude of the 20th EPSC is larger than that of the first (10–71.4 Hz) or similar to that of the first (100 Hz). The effect of GxTX on a train of EPSCs (100 Hz) was also tested (Fig. 6*E*). GxTX had little effect on the EPSC amplitude, and the differences were not significant (two-way repeated measure ANOVA and Bonferroni *post hoc* tests; Fig. 6*Eii*), indicating that GxTX does not affect the ability of parallel fiber axons to conduct high-frequency spikes or the transmitter release from parallel fiber.

To explore how a train of action potentials evoked by parallel fiber stimulation is affected by the blockade of Kv2 channels, recordings were made under current-clamp conditions (Fig. 7). Because the input-output relationship of simple-spiking cells was quite similar to that of complex-spiking cells (Fig. 5*C–E*), the data from both cell types were combined and pooled for the following quantitative analysis (Fig. 7*E*). Spike probability was calculated from five trials, and we defined that successful action potential has maximum rate of rise >30 V/s and peak amplitude higher than −15 mV (Fig. 7*D*). Without GxTX, cartwheel cells showed trains of action potentials with high fidelity at 20–71.4 Hz (Fig. 7*A*,*B*, control). At 100 Hz, a few failures were observed in each trial (Fig. 7*C*, control), but most of the stimuli succeeded in inducing action potentials. At 20 Hz, GxTX had little or no effect on the spike probability when parallel fibers were stimulated (Fig. 7*E*, 20 Hz). At higher stimulus rates, however, the blockade of Kv2 channels induced the failure of action potentials in the middle and late of the train stimulation (Fig. 7*A–C*,*E*, 30.3–100 Hz). Taken together, these data demonstrate that blocking Kv2 channels induces failure of sustained firing evoked by a parallel fiber stimulation frequency dependent manner.

## Discussion

To the best of our knowledge, the present study is the first to demonstrate that Kv2 channels play a crucial role in regulating synaptically induced, repetitive firing in a frequency-dependent manner in neurons. Largely, Kv2.1 and Kv2.2 channels were present on the cell body of cartwheel cells, not on AISs nor dendrites (Figs. 1, 2), and Kv2 current accounted for about one-third of the delayed rectifier K current (Fig. 3). Kv2 contributed to accelerating the repolarizing phase of action potential and hyperpolarizing the potential of ADP (Fig. 4). Furthermore, blocking Kv2 channels induced the failure of action potentials in the middle and late sustained firing evoked by current injection (Fig. 5) or parallel fiber stimulation (Fig. 7).

Although there are some reports demonstrating that Kv2 channels are necessary for sustained tonic firing induced by current injection and that Kv2 plays a role in the prevention of depolarization block (Tong et al., 2013; Liu and Bean, 2014; Hönigsperger et al., 2017), this study is the first one that shows the role of Kv2 channels in action potential generation in response to excitatory synaptic inputs, which are more physiological stimuli compared with current injection. In medial nucleus of the trapezoid body auditory neurons, Kv2.2 channels are highly expressed in AIS, and Kv2.2 is necessary for high-frequency firing (Johnston et al., 2008; Tong et al., 2013). Indeed, cartwheel cells belong to auditory neurons, but Kv2.2 distribution in cartwheel cells we observed was strikingly different from that in medial nucleus of the trapezoid body neurons.

### Limitation of immunofluorescent study in dendrites

The cartwheel cells whose cell bodies exist in the surface of the acute slices (200-μm thickness) were whole-cell patched, and the dendrites were labeled by intracellular infusion of biocytin via patch pipette. After that, the dendrites were visualized by streptavidin-conjugated AF 647. When the dendrites were examined under a confocal microscope, we often observed the dendrites elongate to deep direction, where immunoreactivity of Kv2 were not detected. Therefore, considering that the thickness of the slices and the penetration of antibodies, we had to acquire images of proximal dendrites, which exist close to the surface of the slices. Clear immunoreactivities of Kv2 channels were not observed in the proximal dendrites, but we did not slice again the thick slices for patch-clamp recordings to label distal dendrites with antibodies. Therefore, we cannot rule out the possibility that distal dendrites do express Kv2 channels.

### Localization of Kv2.1, Kv2.2, and ryanodine receptors in the cell bodies

Immunofluorescence studies revealed that in most case both Kv2.1 and Kv2.2 existed on the cell body of cartwheel cells (Fig. 1*B*; Table 2), but the overlap between Kv2.1 and Kv2.2 was not observed frequently (Fig. 1*Cv*), suggesting that not all Kv2.1 channels form heterotetramers with Kv2.2 channels (Kihira et al., 2010), most Kv2.1 or Kv2.2 channels might exist as homotetramers in cartwheel cells. In specific neurons, including CA1 pyramidal neurons and striatal medium spiny neurons, Kv2.1 clusters are juxtaposed to clustered ryanodine receptors, a marker of endoplasmic reticulum (ER; Mandikian et al., 2014). Kv2.1 channels organize ER and plasma membrane (PM) junctions (ER-PM junctions) through an interaction with vesicle-associated membrane protein-associated protein isoform A (VAPA) and VAPB (Johnson et al., 2018; Kirmiz et al., 2018a). We expected that Kv2.1 clusters overlapped with the ryanodine receptor; however, such overlap was not found frequently in cartwheel cells (Fig. 1*Cvi*), suggesting that Kv2.1 channels are not involved in the formation of ER-PM junctions in the cells. Cartwheel cells might lack VAPA and/or VAPB.

### Putative mechanism underlying interrupting of sustained firing by GxTX

The activation kinetics of Kv2 channels reported in superior cervical ganglion neurons and in entorhinal cortex layer II stellate cells are relatively slow, and those observed in this study were similar to those in previous studies (Fig. 3*E*; Liu and Bean, 2014; Hönigsperger et al., 2017). During the sustained depolarization induced by current injection or a train of excitatory synaptic input, Kv2 channels would gradually open and contribute to the repolarization of action potentials. Once Kv2 channels were blocked by GxTX, the membrane potential between action potentials becomes more positive, leading to the accumulation of Na channel inactivation, to the interruption of sustained firing, and to produce depolarization block. However, to confirm this hypothesis, further studies using a model simulation are necessary.

### Implications for auditory function

Currently, there have been few studies reporting the excitability of cartwheel cells *in vivo* (Davis and Young, 1997; Ma and Brenowitz, 2012). In anesthetized cats, the maximum rate of firing in response to the best frequency tone is 74 Hz, and the maximum rate reaches 50–130 Hz in response to somatosensory stimulation conveyed by parallel fibers. This firing frequency is within the range of firing frequency examined in this study. The maximum firing frequency induced by the current injection was ∼100 Hz, and the blockade of Kv2 channels by GxTX suppressed the firing frequency to ∼70 Hz (Fig. 5*E*). Cartwheel cells fired reliably up to 100 Hz in response to parallel fiber stimulation, but reliable firing was not observed with GxTX even at 30-Hz stimulation (Fig. 7*E*). Thus, Kv2 channels in cartwheel cells must be activated *in vivo*, and the activity of Kv2 channels could play an important role in the high-frequency firing of cartwheel cells *in vivo*. As cartwheel cells strongly inhibit principal cells of the DCN (Roberts and Trussell, 2010), the regulation of excitability of cartwheel cells by Kv2 channels might be important for DCN functions such as monaural sound localization and cancelling detection of self-generated sounds (May, 2000; Singla et al., 2017).

## Synthesis

Reviewing Editor: Christian Hansel, University of Chicago Decisions are customarily a result of the Reviewing Editor and the peer reviewers coming together and discussing their recommendations until a consensus is reached. When revisions are invited, a fact-based synthesis statement explaining their decision and outlining what is needed to prepare a revision will be listed below. The following reviewer(s) agreed to reveal their identity: Michael Roberts.

This manuscript provides novel insight into how Kv2 channels support high frequency firing in DCN cartwheel cells. The results address fundamental mechanisms that regulate neuronal excitability and therefore will be of interest to a large portion of the neuroscience community.

The work examines the role of Kv2.x channels in sustained action potential activity of cartwheel cells in dorsal cochlear nucleus, a neuronal class that has emerged as a model for understanding complexities associated with a range of firing types. Cartwheel cells fire a mix of complex and simple spikes, and the conductances underlying these different types of AP output are fairly well established.

Here, the authors show that Kv2.x channels make important contributions to this spiking activity, providing the currents necessary to prevent neurons from firing an excess of bursts and further prevent neurons from entering depolarization block. This is an important advance, as it is one of the most detailed characterizations of the function of Kv2 in a neuron that exhibits complicated, intrinsic AP output patterns.

Major comments:

In this manuscript, the authors examine how Kv2 channels contribute to the excitability of cartwheel cells in the dorsal cochlear nucleus (DCN). The authors first demonstrate that Kv2.1 and Kv2.2 channel subunits are expressed in the somata of cartwheel cells and not on the axon initial segments. This contrasts with MNTB neurons, where Kv2.2 subunits are densely expressed in the AIS. The authors then use current clamp and voltage clamp recordings in brain slices to show that GxTx-sensitive Kv2 conductances are critical for the ability of cartwheel cells to sustain high frequency firing, including high frequency firing elicited by synaptic inputs. The experiments are well-designed and carefully executed, and the results shed interesting light on how Kv2 channels, a significantly understudied member of the Kv family, shape neuronal excitability. However, there are some concerns that need addressed, including issues with replicates in the anatomy experiment, the statistics used in cases where multiple comparisons were made, and some other minor matters.

Overall, the work is well done, with a mix of high resolution confocal imaging of Kv2s and their co-localization with ryanodine receptors or axon initial segment markers (e.g., AnkG), high-quality voltage clamp, and analysis of current clamp spiking activity.

1) It would be quite nice to see more examples of Kv2 staining in cartwheel cells. As it stands, only 1 somata is shown and 2 other initial segments. It would be quite nice to see more zoomed out images, and better analysis across a population of cartwheel cells for the co-localization of Kv2s and RyRs on the somatic membrane. Furthermore, could the authors provide some detail on whether Kv2s were found on cartwheel cell dendrites?

2) Related to this, several groups have shown that VAMP/RyR-associated Kv2 clusters on the soma contain non-conducting Kv2s. This is hinted at in the discussion, but it is worth being more explicit about this aspect of Kv2 function. How can one be confident that the Gtx-sensitive current being recorded here is coming from somatic Kv2s, rather than a more diffuse population, perhaps also expressed on cartwheel cell dendrites? Some discussion of this would be useful (as well as a better examination of a relative lack of overlap between Kv2 and RyR, as this would be one indication that these channels are indeed conducting. Patches pulled from the soma would provide definitive proof, but in the absence of these data, the authors should consider that they may be measuring non-somatic current.

3) Figure 1 and associated text: The authors conclude that Kv2 subunits are found in the somata of cartwheel cells and not in the AIS, but they provide limited evidence to support this. The data shown in Figure 1 appear convincing on their own, but these data represent samples from only three cartwheel cells. Additionally, quantification of results from additional cells is not provided in the text. The authors should expand their analysis to include data from multiple cartwheel cells across at least three mice to test whether their conclusions are supported by a more thorough data set.

4) In the Methods, line 229, the authors state that they used paired t-tests to assess statistical significance. I did not find mention of other statistical tests elsewhere in the text. This is a concern because many of the analyses involved multiple comparisons (for example, Figure 1B, Figure 4C-E, Figure 6D), and it does not appear that p-values were adjusted for this (for example, Bonferroni correction or a more sophisticated approach). The authors should reanalyze these data with appropriate statistical tests and revise the text and conclusions accordingly.

5) Figure 6 and associated text: What criteria were used to determine when there was a spike failure? I did not find the criteria detailed in the Methods or Results. On a related note, since Kv2 channels are found in the soma and not the AIS, isn't it possible that spikes later in a train might still be full spikes in the AIS but appear as failures in the soma due to NaV inactivation localized to the soma? The AIS presumably has other Kv conductances that help maintain the AIS membrane potential during high frequency trains, and therefore action potentials might still be sent down the axon but be unable to fully backpropagate into the soma. The somatic depolarizations marked as failures in Figure 6 look consistent with this interpretation to me. Maybe phase-plane analysis would help to clear this up?

Minor concerns

1. Line 306: The wording in the parenthetical clause is a bit ambiguous. Perhaps revise to, “20 ms *starting* from the depolarization onset.”

2. Line 461: “Acclimation” is probably a typo for “accumulation.”

3. Line 468: Would it be sufficient to state “74 Hz” and drop the decimal? “74.3 Hz” seems more precise than is meaningful.

4. Lines 472 - 473: Again, to make the language a bit clearer, consider replacing “fired well” with “fired reliably” and replacing “but this effect” with “but reliable firing.”

## Author Response

Response to reviewer's comment

This manuscript provides novel insight into how Kv2 channels support high frequency firing in DCN

cartwheel cells. The results address fundamental mechanisms that regulate neuronal excitability and

therefore will be of interest to a large portion of the neuroscience community.

The work examines the role of Kv2.x channels in sustained action potential activity of cartwheel

cells in dorsal cochlear nucleus, a neuronal class that has emerged as a model for understanding

complexities associated with a range of firing types. Cartwheel cells fire a mix of complex and

simple spikes, and the conductances underlying these different types of AP output are fairly well

established.

Here, the authors show that Kv2.x channels make important contributions to this spiking activity,

providing the currents necessary to prevent neurons from firing an excess of bursts and further

prevent neurons from entering depolarization block. This is an important advance, as it is one of the

most detailed characterizations of the function of Kv2 in a neuron that exhibits complicated, intrinsic

AP output patterns.

Response: We are grateful to the reviewer for the critical comments and useful suggestions that have

helped us to improve our paper. As indicated in the responses that follow, we have taken all these

comments and suggestions into account in the revised version of our paper.

Major comments:

In this manuscript, the authors examine how Kv2 channels contribute to the excitability of cartwheel

cells in the dorsal cochlear nucleus (DCN). The authors first demonstrate that Kv2.1 and Kv2.2

channel subunits are expressed in the somata of cartwheel cells and not on the axon initial segments.

This contrasts with MNTB neurons, where Kv2.2 subunits are densely expressed in the AIS. The

authors then use current clamp and voltage clamp recordings in brain slices to show that

GxTx-sensitive Kv2 conductances are critical for the ability of cartwheel cells to sustain high

frequency firing, including high frequency firing elicited by synaptic inputs. The experiments are

well-designed and carefully executed, and the results shed interesting light on how Kv2 channels, a

significantly understudied member of the Kv family, shape neuronal excitability. However, there are

some concerns that need addressed, including issues with replicates in the anatomy experiment, the

statistics used in cases where multiple comparisons were made, and some other minor matters.

Overall, the work is well done, with a mix of high resolution confocal imaging of Kv2s and their

co-localization with ryanodine receptors or axon initial segment markers (e.g., AnkG), high-quality

voltage clamp, and analysis of current clamp spiking activity.2

1) It would be quite nice to see more examples of Kv2 staining in cartwheel cells. As it stands, only

1 somata is shown and 2 other initial segments. It would be quite nice to see more zoomed out

images, and better analysis across a population of cartwheel cells for the co-localization of Kv2s and

RyRs on the somatic membrane. Furthermore, could the authors provide some detail on whether

Kv2s were found on cartwheel cell dendrites?

Response: According to the suggestion, zoomed out images were placed in Fig. 1B, and we also

quantified the immunoreactivity of Kv2 from three mice (Table. 2). We unexpectedly found some of

cartwheel cells are Kv2.1 negative. Almost all of cartwheel cells examined were Kv2.2 positive

(Table. 2). Please look at the cell pointed by arrows in Fig. 1B. In figure 1B, four cells are Kv2.1

positive (arrowheads) but one cell are Kv2.1 negative (arrows). In order to investigate the presence

of Kv2 in dendrites, we performed intracellular labeling using biocytin-Alexa Fluor dye (Fig. 2C).

All proximal dendrites examined were Kv2-negative (Table.2, n=10). We suppose that Kv2 current

we recorded were derived from cell body. Off course, measurement of immunoreactivity in thick

slices (200 m) have the limitation of antibody penetration. Distal dendrite may express Kv2

channels. So we mentioned this concern in Discussion (line 466).

2) Related to this, several groups have shown that VAMP/RyR-associated Kv2 clusters on the soma

contain non-conducting Kv2s. This is hinted at in the discussion, but it is worth being more explicit

about this aspect of Kv2 function. How can one be confident that the Gtx-sensitive current being

recorded here is coming from somatic Kv2s, rather than a more diffuse population, perhaps also

expressed on cartwheel cell dendrites? Some discussion of this would be useful (as well as a better

examination of a relative lack of overlap between Kv2 and RyR, as this would be one indication that

these channels are indeed conducting. Patches pulled from the soma would provide definitive proof,

but in the absence of these data, the authors should consider that they may be measuring non-somatic

current.

Response: We agree with the reviewer’s comment. We performed intracellular labeling combined

with fluorescence immunohistochemistry. We examined 10 biocytin-filled cells whether the

dendrites were labelled with anti Kv2 antibodies. None out of 10 dendrites were immunopositive

(Fig. 2C, Table 2). However, this method has limitation related to antibody penetration. Distal

dendrite may express Kv2 channels. So we mentioned this concern in Discussion (line 466).3

3) Figure 1 and associated text: The authors conclude that Kv2 subunits are found in the somata of

cartwheel cells and not in the AIS, but they provide limited evidence to support this. The data shown

in Figure 1 appear convincing on their own, but these data represent samples from only three

cartwheel cells. Additionally, quantification of results from additional cells is not provided in the text.

The authors should expand their analysis to include data from multiple cartwheel cells across at least

three mice to test whether their conclusions are supported by a more thorough data set.

Response: According to the suggestion, we increased the number of animal (n=3 in total) for the

anatomical experiment. For the quantification, we counted 130 cell bodies, 12 AISs from three mice.

10 proximal dendrites from 10 cells were also examined. Such quantitative data were summarized in

Table. 2.

4) In the Methods, line 229, the authors state that they used paired t-tests to assess statistical

significance. I did not find mention of other statistical tests elsewhere in the text. This is a concern

because many of the analyses involved multiple comparisons (for example, Figure 1B, Figure 4C-E,

Figure 6D), and it does not appear that p-values were adjusted for this (for example, Bonferroni

correction or a more sophisticated approach). The authors should reanalyze these data with

appropriate statistical tests and revise the text and conclusions accordingly.

Response: Firstly, asterisks (*) in Fig. 1C and Fig. 7A-C did not used for statistical significant. We

used as a mark. Therefore, we replaced the asterisks with hash marks (#) to avoid such confusing.

Secondly, we feel that the reviewer’s comments concerning Fig 4C-E (Fig. 5C-5E in revision) result

from a misinterpretation of the data. Please see the supplemental figure pasted below. For example,

in Fig. 5C, we performed paired t-test between the open square with the red circle at 700 pA. These

data pair were obtained in the absence or presence of GxTX. So we suppose paired t-test is

appropriate. After that, we conducted the same test between the open square with the red circle at

650 pA. We suppose that these statistical analysis does not contain multiple comparison. These two

groups must be independent from the pair at 700pA. If our interpretation is wrong, please point out

our misunderstandings in

detail.4

5) Figure 6 and associated text: What criteria were used to determine when there was a spike failure?

I did not find the criteria detailed in the Methods or Results. On a related note, since Kv2 channels

are found in the soma and not the AIS, isn’t it possible that spikes later in a train might still be full

spikes in the AIS but appear as failures in the soma due to Nav inactivation localized to the soma?

The AIS presumably has other Kv conductances that help maintain the AIS membrane potential

during high frequency trains, and therefore action potentials might still be sent down the axon but be

unable to fully backpropagate into the soma. The somatic depolarizations marked as failures in

Figure 6 look consistent with this interpretation to me. Maybe phase-plane analysis would help to

clear this up?

Response: According to the reviewer’s comment, we defined spike failure as follows. Firstly, we

differentiated six traces showing 20 synaptic responses at 100 Hz in the presence or absence of

GxTX (e.g. Fig. 7Cii). Then we plotted on a semi-logarithmic scale the relationship of the maximum

rate of rise and the rate of decay (Fig. 7D). Finally, with the aid of the plot, we defined the spike

failure as depolarization which had the maximum rate of spike lower than 100 v/s (dashed line in Fig.

7Cii and 7D). We then recalculated spike failures following the criteria and replotted in Fig. 7E.

Minor concerns

1. Line 306: The wording in the parenthetical clause is a bit ambiguous. Perhaps revise to, “20 ms

*starting* from the depolarization onset.”

Response: In accordance with the reviewer’s recommendation, “starting” was inserted in the

sentence (line 333).

2. Line 461: “Acclimation” is probably a typo for “accumulation.”

Response: The typographical error has been addressed in the revised manuscript (line 501).

3. Line 468: Would it be sufficient to state “74 Hz” and drop the decimal? “74.3 Hz” seems more

precise than is meaningful.

Response: In accordance with the reviewer’s recommendation, we deleted “.3” (line 508).5

4. Lines 472 - 473: Again, to make the language a bit clearer, consider replacing “fired well” with

"fired reliably” and replacing “but this effect” with “but reliable firing.”

Response: In accordance with the reviewer’s recommendation, the replacements were done (line

512-513)

## References

[B1] Bender KJ, Uebele VN, Renger JJ, Trussell LO (2012) Control of firing patterns through modulation of axon initial segment T-type calcium channels. J Physiol 590:109–118. 10.1113/jphysiol.2011.218768 22063631 PMC3300050

[B2] Bishop HI, Guan D, Bocksteins E, Parajuli LK, Murray KD, Cobb MM, Misonou H, Zito K, Foehring RC, Trimmer JS (2015) Distinct cell- and layer-specific expression patterns and independent regulation of Kv2 channel subtypes in cortical pyramidal neurons. J Neurosci 35:14922–14942. 10.1523/JNEUROSCI.1897-15.2015 26538660 PMC4635138

[B3] Davis KA, Young ED (1997) Granule cell activation of complex-spiking neurons in dorsal cochlear nucleus. J Neurosci 17:6798–6806. 9254690 10.1523/JNEUROSCI.17-17-06798.1997 PMC6573148

[B4] Golding NL, Oertel D (1996) Context-dependent synaptic action of glycinergic and GABAergic inputs in the dorsal cochlear nucleus. J Neurosci 16:2208–2219. 8601801 10.1523/JNEUROSCI.16-07-02208.1996 PMC6578533

[B5] Golding NL, Oertel D (1997) Physiological identification of the targets of cartwheel cells in the dorsal cochlear nucleus. J Neurophysiol 78:248–260. 10.1152/jn.1997.78.1.248 9242277

[B6] Guan D, Tkatch T, Surmeier DJ, Armstrong WE, Foehring RC (2007) Kv2 subunits underlie slowly inactivating potassium current in rat neocortical pyramidal neurons. J Physiol 581:941–960. 10.1113/jphysiol.2007.128454 17379638 PMC2170822

[B7] Herrington J (2007) Gating modifier peptides as probes of pancreatic beta-cell physiology. Toxicon 49:231–238. 10.1016/j.toxicon.2006.09.012 17101164

[B8] Herrington J, Zhou YP, Bugianesi RM, Dulski PM, Feng Y, Warren VA, Smith MM, Kohler MG, Garsky VM, Sanchez M, Wagner M, Raphaelli K, Banerjee P, Ahaghotu C, Wunderler D, Priest BT, Mehl JT, Garcia ML, McManus OB, Kaczorowski GJ, Slaughter RS (2006) Blockers of the delayed-rectifier potassium current in pancreatic beta-cells enhance glucose-dependent insulin secretion. Diabetes 55:1034–1042. 10.2337/diabetes.55.04.06.db05-0788 16567526

[B9] Hille B (2001) Ion channels of excitable membranes, Ed 3. Sunderland: Sinauer.

[B10] Hönigsperger C, Nigro MJ, Storm JF (2017) Physiological roles of Kv2 channels in entorhinal cortex layer II stellate cells revealed by Guangxitoxin-1E. J Physiol 595:739–757. 10.1113/JP273024 27562026 PMC5285721

[B11] Hwang PM, Fotuhi M, Bredt DS, Cunningham AM, Snyder SH (1993) Contrasting immunohistochemical localizations in rat brain of two novel K+ channels of the Shab subfamily. J Neurosci 13:1569–1576. 8463836 10.1523/JNEUROSCI.13-04-01569.1993 PMC6576723

[B12] Irie T, Trussell LO (2017) Double-nanodomain coupling of calcium channels, ryanodine receptors, and BK channels controls the generation of burst firing. Neuron 96:856–870.e4. 10.1016/j.neuron.2017.10.014 29144974 PMC5758055

[B13] Johnson B, Leek AN, Solé L, Maverick EE, Levine TP, Tamkun MM (2018) Kv2 potassium channels form endoplasmic reticulum/plasma membrane junctions via interaction with VAPA and VAPB. Proc Natl Acad Sci USA 115:E7331–E7340. 10.1073/pnas.1805757115 29941597 PMC6077746

[B14] Johnson B, Leek AN, Tamkun MM (2019) Kv2 channels create endoplasmic reticulum/plasma membrane junctions: a brief history of Kv2 channel subcellular localization. Channels (Austin) 13:88–101. 10.1080/19336950.2019.1568824 30712450 PMC6380216

[B15] Johnston J, Griffin SJ, Baker C, Skrzypiec A, Chernova T, Forsythe ID (2008) Initial segment Kv2.2 channels mediate a slow delayed rectifier and maintain high frequency action potential firing in medial nucleus of the trapezoid body neurons. J Physiol 586:3493–3509. 10.1113/jphysiol.2008.153734 18511484 PMC2538803

[B16] Johnston J, Forsythe ID, Kopp-Scheinpflug C (2010) Going native: voltage-gated potassium channels controlling neuronal excitability. J Physiol 588:3187–3200. 10.1113/jphysiol.2010.191973 20519310 PMC2976014

[B17] Kihira Y, Hermanstyne TO, Misonou H (2010) Formation of heteromeric Kv2 channels in mammalian brain neurons. J Biol Chem 285:15048–15055. 10.1074/jbc.M109.074260 20202934 PMC2865335

[B18] Kim Y, Trussell LO (2007) Ion channels generating complex spikes in cartwheel cells of the dorsal cochlear nucleus. J Neurophysiol 97:1705–1725. 10.1152/jn.00536.2006 17289937

[B19] Kimm T, Khaliq ZM, Bean BP (2015) Differential regulation of action potential shape and burst-frequency firing by BK and Kv2 channels in substantia nigra dopaminergic neurons. J Neurosci 35:16404–16417. 10.1523/JNEUROSCI.5291-14.2015 26674866 PMC4679822

[B20] King AN, Manning CF, Trimmer JS (2014) A unique ion channel clustering domain on the axon initial segment of mammalian neurons. J Comp Neurol 522:2594–2608. 10.1002/cne.23551 24477962 PMC4133991

[B21] Kirmiz M, Vierra NC, Palacio S, Trimmer JS (2018a) Identification of VAPA and VAPB as Kv2 channel-interacting proteins defining endoplasmic reticulum-plasma membrane junctions in mammalian brain neurons. J Neurosci 38:7562–7584. 10.1523/JNEUROSCI.0893-18.2018 30012696 PMC6113906

[B22] Kirmiz M, Palacio S, Thapa P, King AN, Sack JT, Trimmer JS (2018b) Remodeling neuronal ER-PM junctions is a conserved nonconducting function of Kv2 plasma membrane ion channels. Mol Biol Cell 29:2410–2432. 10.1091/mbc.E18-05-0337 30091655 PMC6233057

[B23] Kole MH, Stuart GJ (2012) Signal processing in the axon initial segment. Neuron 73:235–247. 10.1016/j.neuron.2012.01.007 22284179

[B24] Liu PW, Bean BP (2014) Kv2 channel regulation of action potential repolarization and firing patterns in superior cervical ganglion neurons and hippocampal CA1 pyramidal neurons. J Neurosci 34:4991–5002. 10.1523/JNEUROSCI.1925-13.2014 24695716 PMC3972724

[B25] Ma WL, Brenowitz SD (2012) Single-neuron recordings from unanesthetized mouse dorsal cochlear nucleus. J Neurophysiol 107:824–835. 10.1152/jn.00427.2011 22072506 PMC3289476

[B26] Malin SA, Nerbonne JM (2002) Delayed rectifier K+ currents, IK, are encoded by Kv2 alpha-subunits and regulate tonic firing in mammalian sympathetic neurons. J Neurosci 22:10094–10105. 12451110 10.1523/JNEUROSCI.22-23-10094.2002 PMC6758768

[B27] Mandikian D, Bocksteins E, Parajuli LK, Bishop HI, Cerda O, Shigemoto R, Trimmer JS (2014) Cell type-specific spatial and functional coupling between mammalian brain Kv2.1 K+ channels and ryanodine receptors. J Comp Neurol 522:3555–3574. 10.1002/cne.23641 24962901 PMC4139460

[B28] Manis PB, Spirou GA, Wright DD, Paydar S, Ryugo DK (1994) Physiology and morphology of complex spiking neurons in the guinea pig dorsal cochlear nucleus. J Comp Neurol 348:261–276. 10.1002/cne.903480208 7814691

[B29] May BJ (2000) Role of the dorsal cochlear nucleus in the sound localization behavior of cats. Hear Res 148:74–87. 10.1016/s0378-5955(00)00142-8 10978826

[B30] Murakoshi H, Trimmer JS (1999) Identification of the Kv2.1 K+ channel as a major component of the delayed rectifier K+ current in rat hippocampal neurons. J Neurosci 19:1728–1735. 10.1523/JNEUROSCI.19-05-01728.1999 10024359 PMC6782166

[B31] Oertel D, Young ED (2004) What’s a cerebellar circuit doing in the auditory system? Trends Neurosci 27:104–110. 10.1016/j.tins.2003.12.001 15102490

[B32] Roberts MT, Trussell LO (2010) Molecular layer inhibitory interneurons provide feedforward and lateral inhibition in the dorsal cochlear nucleus. J Neurophysiol 104:2462–2473. 10.1152/jn.00312.2010 20719922 PMC2997026

[B33] Roberts MT, Bender KJ, Trussell LO (2008) Fidelity of complex spike-mediated synaptic transmission between inhibitory interneurons. J Neurosci 28:9440–9450. 10.1523/JNEUROSCI.2226-08.2008 18799676 PMC2628470

[B34] Rothman JS, Silver RA (2018) NeuroMatic: an integrated open-source software toolkit for acquisition, analysis and simulation of electrophysiological data. Front Neuroinform 12:14. 10.3389/fninf.2018.00014 29670519 PMC5893720

[B35] Singla S, Dempsey C, Warren R, Enikolopov AG, Sawtell NB (2017) A cerebellum-like circuit in the auditory system cancels responses to self-generated sounds. Nat Neurosci 20:943–950. 10.1038/nn.4567 28530663 PMC5525154

[B36] Tong H, Kopp-Scheinpflug C, Pilati N, Robinson SW, Sinclair JL, Steinert JR, Barnes-Davies M, Allfree R, Grubb BD, Young SM Jr, Forsythe ID (2013) Protection from noise-induced hearing loss by Kv2.2 potassium currents in the central medial olivocochlear system. J Neurosci 33:9113–9121. 10.1523/JNEUROSCI.5043-12.2013 23699522 PMC5503134

[B37] Trimmer JS (1991) Immunological identification and characterization of a delayed rectifier K+ channel polypeptide in rat brain. Proc Natl Acad Sci USA 88:10764–10768. 10.1073/pnas.88.23.10764 1961744 PMC53011

[B38] Tzounopoulos T, Kim Y, Oertel D, Trussell LO (2004) Cell-specific, spike timing-dependent plasticities in the dorsal cochlear nucleus. Nat Neurosci 7:719–725. 10.1038/nn1272 15208632

[B39] Vacher H, Mohapatra DP, Trimmer JS (2008) Localization and targeting of voltage-dependent ion channels in mammalian central neurons. Physiol Rev 88:1407–1447. 10.1152/physrev.00002.2008 18923186 PMC2587220

[B40] Vogler NW, Betti VM, Goldberg JM, Tzounopoulos T (2020) Mechanisms underlying long-term synaptic zinc plasticity at mouse dorsal cochlear nucleus glutamatergic synapses. J Neurosci 40:4981–4996. 10.1523/JNEUROSCI.0175-20.2020 32434779 PMC7314415

[B41] Wouterlood FG, Mugnaini E (1984) Cartwheel neurons of the dorsal cochlear nucleus: a Golgi-electron microscopic study in rat. J Comp Neurol 227:136–157. 10.1002/cne.902270114 6088594

[B42] Yang S, Ben-Shalom R, Ahn M, Liptak AT, van Rijn RM, Whistler JL, Bender KJ (2016) β-Arrestin-dependent dopaminergic regulation of calcium channel activity in the axon initial segment. Cell Rep 16:1518–1526. 10.1016/j.celrep.2016.06.098 27452469 PMC5074334

